# A genomics approach to identify susceptibilities of breast cancer cells to “fever-range” hyperthermia

**DOI:** 10.1186/1471-2407-14-81

**Published:** 2014-02-11

**Authors:** Clarissa Amaya, Vittal Kurisetty, Jessica Stiles, Alice M Nyakeriga, Arunkumar Arumugam, Rajkumar Lakshmanaswamy, Cristian E Botez, Dianne C Mitchell, Brad A Bryan

**Affiliations:** 1 Department of Biomedical Sciences, Paul L. Foster School of Medicine, Texas Tech University Health Sciences Center, 5001 El Paso Drive, MSB1 Room 2111, El Paso, Texas 79905, USA; 2 Department of Physics, University of Texas, El Paso, Texas USA

**Keywords:** Breast cancer, Hyperthermia, Heat shock, Microarray, Genomics, Gene expression

## Abstract

**Background:**

Preclinical and clinical studies have shown for decades that tumor cells demonstrate significantly enhanced sensitivity to “fever range” hyperthermia (increasing the intratumoral temperature to 42-45°C) than normal cells, although it is unknown why cancer cells exhibit this distinctive susceptibility.

**Methods:**

To address this issue, mammary epithelial cells and three malignant breast cancer lines were subjected to hyperthermic shock and microarray, bioinformatics, and network analysis of the global transcription changes was subsequently performed.

**Results:**

Bioinformatics analysis differentiated the gene expression patterns that distinguish the heat shock response of normal cells from malignant breast cancer cells, revealing that the gene expression profiles of mammary epithelial cells are completely distinct from malignant breast cancer lines following this treatment. Using gene network analysis, we identified altered expression of transcripts involved in mitotic regulators, histones, and non-protein coding RNAs as the significant processes that differed between the hyperthermic response of mammary epithelial cells and breast cancer cells. We confirmed our data via qPCR and flow cytometric analysis to demonstrate that hyperthermia specifically disrupts the expression of key mitotic regulators and G2/M phase progression in the breast cancer cells.

**Conclusion:**

These data have identified molecular mechanisms by which breast cancer lines may exhibit enhanced susceptibility to hyperthermic shock.

## Background

Although the effectiveness of standard therapies such as surgery, chemotherapy, and irradiation has steadily improved over the years, cancer remains one of the most challenging problems of modern medicine. Among the major issues that complicate cancer treatment is the fact that cancerous cells are very difficult to therapeutically target with any specificity as they are in many respects similar to normal cells and have an astonishing ability of “hiding” their peculiarities. It has been known for over three decades that tumor cells demonstrate significantly more sensitivity to mild hyperthermia in “fever-range” temperatures (41-45°C) than normal cells [1,2]. Mild hyperthermia has been shown in a wealth of preclinical oncology studies to act as a dose modifying agent that increases the therapeutic ratio of conventional therapy, thus enhancing the effectiveness of a given dose without additional toxicity [3]. Furthermore, numerous clinical trials have combined hyperthermia with radiation therapy and/or chemotherapy for many types of carcinomas (including breast cancers) and sarcomas, and most studies have shown a significant reduction in tumor volume when hyperthermia is combined with standard treatments [4-7]. Various hyperthermia techniques have been developed to treat breast cancer, including focused ultrasound [8], focused microwaves [9,10], and radiofrequency electric fields [11]. Despite these techniques, various factors including tumor size and depth greatly affect the homogenous distribution of heat specific to and throughout the entire tumor mass. With the recent and rapid progression of nanobiotechnology applications in medicine, the development of magnetic nanoparticles which can induce tumor hyperthermia through hysteresis loss in an alternating magnetic field has renewed great interest in reexamining this adjuvant therapy in tumor treatments [12]. Further development of this technology may have the potential to overcome the previous limitations associated with older modalities of inducing hyperthermia and lead to reduced morbidity and mortality for patients.

Hyperthermia over a short period (generally 30 minutes to 1 hour) has been shown to induce irreversible cell damage and subsequent death in tumor cells, yet normal cells are remarkably spared [1,2]. These effects are often very rapid, with tumor apoptosis and necrosis occurring within a short time (3–6 hrs) post heating [13]. Several mechanisms have been proposed as to how hyperthermia kills tumor cells including disruption of plasma membrane protein and cytoskeletal distribution, altering mitochondrial membrane potential and cellular redox status, disrupting cell cycle progression, inducing tumor hypoxia, and affecting DNA damage repair mechanisms in the nucleus [14-16], yet despite several decades of research the definitive identification of mechanisms leading to the favorable clinical results of hyperthermia have not been established. It has been further hypothesized that the strong anti-tumor effect of hyperthermia may be due to the low blood flow rate (and thus reduced dispersant cooling following heating) found in the center of tumors due to a disorganized and often dysfunctional vascular system. Additionally, several reports indicate that hyperthermia induces a strong immunological response via activation of immune cells and sensitization of tumor cells to immune effector cells [17-19]. Several studies have elucidated the heat shock induced changes in global gene expression of tumor cell lines such as squamous cell carcinoma, lymphoma, and glioma and have commonly identified gene networks involved in apoptosis, cell cycle, and cell structure/maintanence [20-22]. However, none of these studies compared the gene expression profiles to that of hyperthermia treated normal cells, thus it remains unknown how the hyperthermic response of cancer cells differs from that of normal cells. Identification of the unique hyperthermia-induced gene expression changes between normal and cancer cells may not only shed light on the selective disadvantage of solid tumors in response to mild increases in temperature, but could also identify signaling targets and biological processes which potentially could be exploited to sensitize tumors to chemotherapy and radiation.

To address this issue, we analyzed the hyperthermia-induced global gene expression profiles of a panel of breast cancer and mammary epithelial cell lines and used bioinformatics analysis to identify the unique gene networks distinct between the normal and cancer lines following this treatment. Furthermore, we confirmed our identified gene expression changes using qPCR and utilized flow cytometry to verify that these transcriptional alterations indeed reflect breast cancer specific responses to hyperthermia.

## Methods

### Cell culture and hyperthermia treatment

MCF10A (ATCC #CRL10318) mammary epithelial cells and MCF7 luminal breast cancer cells (ATCC #HTB-22), MDA-MB-231 Basal B breast cancer cells (ATCC # HTB-26), and MDA-MB-468 Basal A breast cancer cells (ATCC #HTB-132) were purchased from ATCC and grown in standard culture conditions as previously reported [23-25]. For heat shock, cells were split into two groups: 37°C control (*C* and *C’* for mammary epithelial and breast cancer cells, respectively) and 45°C hyperthermic treatment (*H* and *H’* for mammary epithelial and breast cancer cells, respectively). The 37°C control was grown under standard culture conditions. For the hyperthermia treatment, 45°C prewarmed conditioned media was immediately added to each treatment group and continuously maintained at this temperature for 30 minutes. After this time, the 45°C media was completely removed and replaced with 37°C conditioned media. The cells were then grown under standard culture conditions and harvested at the time point indicated for each experiment.

### Microarray analysis

Total RNA was collected from each cell line (triplicate biological replicates) 4 hours after completion of the hyperthermia treatment. RNA was amplified and biotin-labeled using Illumina TotalPrep RNA Amplification Kit (Ambion). 750 ng of biotinylated aRNA was then briefly heat-denatured and loaded onto expression arrays to hybridize overnight (triplicate technical replicates). Following hybridization, arrays were labeled with Cy3-streptavidin and imaged on the Illumina ISCAN. Intensity values were transferred to GeneSpring GX microarray analysis software (Agilent) and data was filtered based on quality of each call. Statistical relevance was determined using ANOVA with a Benjamini Hochberg FDR multiple testing correction (p-value < 0.05). Data were then limited by fold change analysis to statistically relevant data points demonstrating a 2-fold or more change in expression. The microarray data from this experiment is publically available on the Gene Expression Omnibus (GEO Accession #GSE48398). All heatmaps shown represent the combined average of all biological and technical replicates.

### Bioinformatics analysis of microarray data

Pathway analysis to identify gene networks and biological processes affected by the gene expression changes was performed using Metacore software (Thomson Reuters). Protein-protein interaction networks were determined using String 9.05 (http://string-db.org).

### Quantitative real time PCR analysis

RNA was isolated from cells 4 hours after the hyperthermia treatment using the Ambion Purelink Minikit according to the manufacturer’s directions. The RNA collected was from an independent biological experiment separate from the RNA collected for the microarray to minimize the discovery of false positives. qRT-PCR was performed on an ABI7900HT RT-PCR system using TaqMan Assays with predesigned primer sets for the genes of interest (Invitrogen). All RT-PCR experiments were performed in at least triplicate.

### Flow cytometry

Cells were harvested 24 hours post treatment via trypsinization and stained with propidium iodide as previous reported [26]. Cell cycle profiles were independently obtained using either a BD LSRII flow cytometer or an Accuri C6 flow cytometer. Flow cytometry data was analyzed using FlowJo software (Tree Star) or CFlow Plus software (Accuri).

## Results

### Determination of the global transcriptional response of mammary epithelial and breast cancer cells to fever range hyperthermia

**Figure 1:**
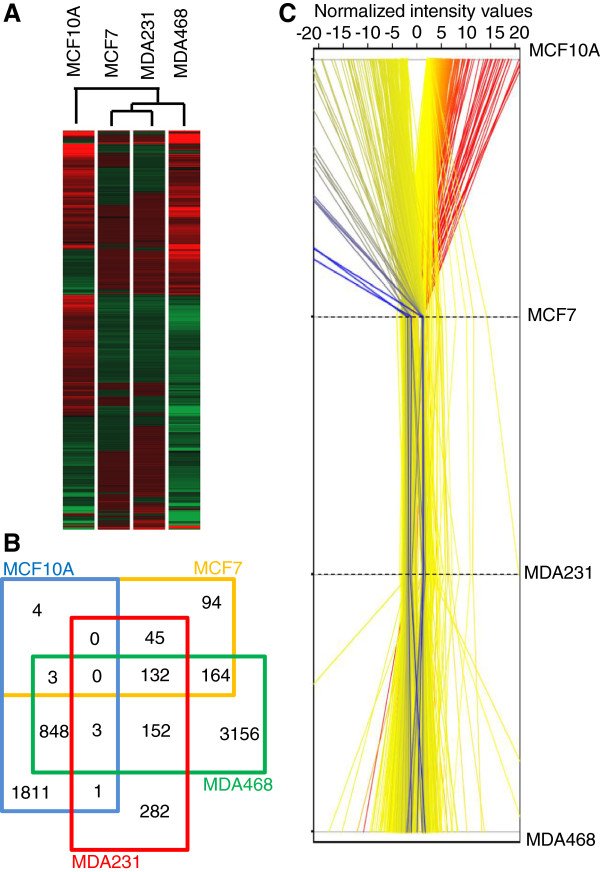
**Fever range hyperthermic shock induces large-scale changes in gene expression in breast cancer and mammary epithelial cells. (A)** Heatmap depicting the 7252 two-fold or greater changes in gene expression (p < 0.05) occurring in the *C* vs *H* and *C’* vs *H’* comparisons. Hierarchical clustering based on cell lines shows the degree of similarity with respect to gene expression clustering for each indicated cell line (*red?=?overexpressed, green?=?underexpressed*). **(B)** Venn diagram illustrating common and unique 2-fold or greater gene expression changes (p < 0.05) between each of the cell lines in the *C* vs *H* and *C’* vs *H’* comparison. **(C)** Profile plot of the normalized intensity values for each two-fold or greater gene expression change (p < 0.05) showing relative expression for each cell line in the in the *C* vs *H* and *C’* vs *H’* comparison.

It remains to be determined how mild hyperthermia preferentially selects against breast cancer cells, yet largely spares normal tissue from collateral damage. To address this question, we first sought to elucidate how hyperthermia induces alterations in gene expression patterns in mammary epithelial and breast cancer cells. Mammary epithelial cells (MCF10A) and three malignant breast cancer lines from each of the known subtypes (MCF7 [luminal], MDA231 [Basal B], and MDA468 [Basal A]) were subjected to 30 minutes of fever range hyperthermic shock (or maintained at 37°C as a control) as described in the Materials and Methods section. To streamline identification of these treatment groups, cells grown at 37°C will be referred to as *C* and *C’* (for mammary epithelial and breast cancer cells, respectively), while cells grown at 45°C will be referred to as *H* and *H’* (for mammary epithelial and breast cancer cells, respectively). Total RNA was isolated 4 hours following hyperthermic treatment. We then performed microarray analysis of the global transcription changes using Illumina high density BeadArrays which measure the expression levels of more than 47,000 transcripts and known splice variants across the human transcriptome. Data was filtered based on quality of each call and statistical relevance was determined using ANOVA with a Benjamini Hochberg FDR multiple testing correction (p-value < 0.05). Data were then limited by fold change analysis to statistically relevant data points demonstrating a 2-fold or more change in expression. When comparing the expression changes based on the *C* vs *H* and *C’* vs *H’* analysis, we discovered that hyperthermia induced very dramatic changes in gene expression in all cell lines tested as reflected by 7252 two-fold or greater statistically significant gene expression changes (p < 0.05) occurring in at least one of the four cell lines (Figure 1A). Specifically, hyperthermia significantly altered the expression of 2670 genes in the MCF10A line (1810 genes upregulated and 860 genes downregulated), 442 genes in MCF7 (72 genes upregulated and 370 genes downregulated), 615 genes in MDA231 (244 genes upregulated and 371 downregulated), and 4458 genes in MDA468 (1744 genes upregulated and 2714 genes downregulated). A list of the top and bottom most regulated genes for each cell line can be found in Table 1. The complete gene expression dataset has been freely and publically deposited in Gene Expression Omnibus for ease of access and meta-analysis (GEO Accession #48398). These data suggest that mild hyperthermia induces large-scale alterations in gene expression profiles across normal and breast cancer cell lines.

**Table 1:** **List of the top and bottom most regulated genes for each cell line in the *C *** **vs *H *** **and *C’ *** **vs *H’ *** **comparisons**

**Gene symbol**	**Gene name**	**Accession number**	**MCF-10A**	**MCF-7**	**MDA-231**	**MDA-468**
MCF10A						
XAGE1A	X antigen family, member 1A, TV3	NM_001097593.1	64.0	1.2	1.0	122.0
XAGE1B	X antigen family, member 1B, TV1	NM_001097595.1	60.3	1.2	1.1	131.6
SRGN	Serglycin, TV1	NM_002727.2	58.1	1.1	-1.5	65.8
SRGN	Serglycin, TV1	NM_002727.2	56.5	1.2	-1.5	67.7
LOC652683	Similar to sperm protein associated with the nucleus, X chromosome, family member B1	XM_942283.2	50.0	1.2	-1.0	71.5
SPANXA2	SPANX family, member A2	NM_145662.2	49.3	1.2	-1.1	67.7
SPANXB1	SPANX family, member B1	NM_032461.2	48.7	1.2	-1.0	70.0
TOP2A	Topoisomerase (DNA) II alpha 170 kDa	NM_001067.2	39.0	-2.0	-3.7	1.1
LOC653219	Similar to G antigen, family D, 2 isoform 1a, TV1	XM_927237.1	37.0	1.2	1.1	71.8
LOC100133171	Hypothetical protein LOC100133171	XM_001713741.1	34.8	1.3	-1.1	52.7
RN5S9	RNA, 5S ribosomal 9	NR_023371.1	-48.2	11.5	59.7	11.5
RN7SK	RNA, 7SK small nuclear	NR_001445.1	-55.1	13.1	45.4	38.6
SERPINB5	Serpin peptidase inhibitor, clade B (ovalbumin), member 5	NM_002639.3	-65.8	1.2	7.6	-3.8
KIAA1666	KIAA1666 protein	XM_942124.2	-72.9	17.9	33.4	35.3
AKR1C2	Aldo-keto reductase family 1, member C2, TV1	NM_001354.4	-82.3	1.4	24.4	-9.6
LOC650517	Hypothetical LOC650517	XR_019109.1	-90.0	1.2	1.7	-2.0
RN7SK	RNA, 7SK small nuclear	NR_001445.1	-109.9	20.2	62.1	45.2
KRT17P3	Predicted misc_RNA KRT17P3	XR_015626.2	-133.2	1.5	2.6	-4.5
LOC651397	Predicted misc_RNA LOC651397	XR_037048.1	-141.4	1.2	5.1	-4.1
KRT6A	Keratin 6A	NM_005554.3	-242.8	1.2	116.7	-11.7
MCF7						
RNY5	RNA, Ro-associated Y5	NR_001571.2	-17.4	22.6	4.6	4.3
RN7SK	RNA, 7SK small nuclear	NR_001445.1	-109.9	20.2	62.1	45.2
KIAA1666	KIAA1666 protein	XM_942124.2	-72.9	17.9	33.4	35.3
MIR1974	MicroRNA 1974	NR_031738.1	-9.4	14.2	20.8	133.3
LOC100008589	28S ribosomal RNA	NR_003287.1	-26.8	13.8	26.4	113.8
RN7SK	RNA, 7SK small nuclear	NR_001445.1	-55.1	13.1	45.4	38.6
LOC100132394	Hypothetical protein LOC100132394	XM_001713809.1	-19.4	12.3	22.2	105.0
RNU4-1	RNA, U4 small nuclear 1	NR_003925.1	-7.7	11.5	11.4	13.2
RN5S9	RNA, 5S ribosomal 9	NR_023371.1	-48.2	11.5	59.7	11.5
SNORD3C	Small nucleolar RNA, C/D box 3C	NR_006881.1	-34.9	11.1	30.5	11.8
CCDC117	Coiled-coil domain containing 117	NM_173510.1	3.2	-3.3	-1.7	-1.8
LOC730432	Hypothetical protein LOC730432	XM_001125680.1	3.7	-3.3	-1.3	-4.7
LOC644799	Hypothetical protein LOC644799, TV1	XM_934554.1	2.5	-3.3	-2.1	1.1
RPS6KB1	Ribosomal protein S6 kinase, 70 kDa, polypeptide 1	NM_003161.2	2.9	-3.4	-1.8	-3.7
NRIP1	Nuclear receptor interacting protein 1	NM_003489.2	1.1	-3.4	-1.8	-4.2
AP4E1	Adaptor-related protein complex 4, epsilon 1 subunit	NM_007347.3	2.3	-3.4	-2.1	-2.2
DCP2	DCP2 decapping enzyme homolog (S. cerevisiae), TV1	NM_152624.4	3.1	-3.4	-2.4	-6.8
TROVE2	TROVE domain family, member 2, TV1	NM_001042369.1	2.0	-3.8	-1.2	-8.2
PURB	Purine-rich element binding protein B	NM_033224.3	3.2	-3.8	-2.6	-7.1
ZNF217	Zinc finger protein 217	NM_006526.2	3.2	-3.9	-1.7	-2.5
MDA231						
KRT6A	Keratin 6A	NM_005554.3	-242.8	1.2	116.7	-11.7
RN7SK	RNA, 7SK small nuclear	NR_001445.1	-109.9	20.2	62.1	45.2
RN5S9	RNA, 5S ribosomal 9	NR_023371.1	-48.2	11.5	59.7	11.5
RN7SK	RNA, 7SK small nuclear	NR_001445.1	-55.1	13.1	45.4	38.6
SNORD3D	Small nucleolar RNA, C/D box 3D	NR_006882.1	-47.2	11.1	36.8	9.4
SNORD3A	Small nucleolar RNA, C/D box 3A	NR_006880.1	-46.0	11.1	36.5	9.0
KIAA1666	KIAA1666 protein	XM_942124.2	-72.9	17.9	33.4	35.3
SNORD3C	Small nucleolar RNA, C/D box 3C	NR_006881.1	-34.9	11.1	30.5	11.8
LOC100008589	28S ribosomal RNA	NR_003287.1	-26.8	13.8	26.4	113.8
LOC100132564	Hypothetical protein LOC100132564	XM_001713808.1	-21.6	8.4	25.3	13.3
PAK2	p21 protein (Cdc42/Rac)-activated kinase 2	NM_002577.3	5.9	-2.9	-3.1	-1.3
CEP55	Centrosomal protein 55 kDa, TV1	NM_018131.3	19.2	-1.7	-3.2	-1.2
RHOBTB3	Rho-related BTB domain containing 3	NM_014899.3	14.0	-1.1	-3.2	2.5
ZAK	Sterile alpha motif and leucine zipper containing kinase AZK, V2	NM_133646.2	2.8	-2.6	-3.2	-4.0
KATNAL1	Katanin p60 subunit A-like 1, TV2	NM_001014380.1	3.6	-1.4	-3.2	-1.3
PBK	PDZ binding kinase	NM_018492.2	10.5	-1.9	-3.3	-1.0
PAFAH1B1	Platelet-activating factor acetylhydrolase, isoform Ib, alpha subunit	NM_000430.2	4.5	-2.6	-3.5	1.2
TOP2A	Topoisomerase (DNA) II alpha 170 kDa	NM_001067.2	39.0	-2.0	-3.7	1.1
FAM83D	Family with sequence similarity 83, member D	NM_030919.2	15.4	-1.8	-4.0	-1.7
BCAT1	Branched chain aminotransferase 1, cytosolic	NM_005504.4	8.9	1.1	-4.9	2.9
MDA468						
MIR1974	microRNA 1974	NR_031738.1	-9.4	14.2	20.8	133.3
XAGE1B	X antigen family, member 1B, TV1	NM_001097595.1	60.3	1.2	1.1	131.6
XAGE1A	X antigen family, member 1A, TV3	NM_001097593.1	64.0	1.2	1.0	122.0
LOC100008589	28S ribosomal RNA	NR_003287.1	-26.8	13.8	26.4	113.8
LOC100132394	Hypothetical protein LOC100132394	XM_001713809.1	-19.4	12.3	22.2	105.0
CST3	Cystatin C	NM_000099.2	-1.8	1.2	1.7	103.9
ACTG1	Actin, gamma 1	NM_001614.2	1.1	1.5	1.5	75.2
LOC653219	Similar to G antigen, family D, 2 isoform 1a,	XM_927237.1	37.0	1.2	1.1	71.8
LOC652683	Similar to sperm protein associated with the nucleus, X chromosome, family member B1	XM_942283.2	50.0	1.2	-1.0	71.5
SPANXB1	SPANX family, member B1	NM_032461.2	48.7	1.2	-1.0	70.0
CSAG1	Chondrosarcoma associated gene 1, TVb	NM_153479.1	1.7	1.2	-1.0	-27.4
SERPINA3	Serpin peptidase inhibitor, clade A (alpha-1 antiproteinase, antitrypsin), member 3	NM_001085.4	-8.5	1.1	2.0	-30.5
ALDH1A3	Aldehyde dehydrogenase 1 family, member A3	NM_000693.1	-1.5	-1.0	2.0	-34.0
EPCAM	Epithelial cell adhesion molecule	NM_002354.2	1.6	-1.2	-1.2	-36.1
LAD1	Ladinin 1	NM_005558.3	-47.9	1.2	13.5	-37.5
OLFML3	Olfactomedin-like 3	NM_020190.2	-1.2	1.2	1.2	-39.0
TACSTD1	Tumor-associated calcium signal transducer 1	NM_002354.1	2.1	-1.1	-1.4	-47.5
RARRES1	Retinoic acid receptor responder (tazarotene induced) 1, TV1	NM_206963.1	-1.3	1.3	-1.1	-49.9
MGP	Matrix Gla protein	NM_000900.2	-18.6	1.0	2.4	-51.1
KLK5	Kallikrein-related peptidase 5, TV1	NM_012427.4	-1.3	1.1	1.1	-56.3

**Figure 2:**
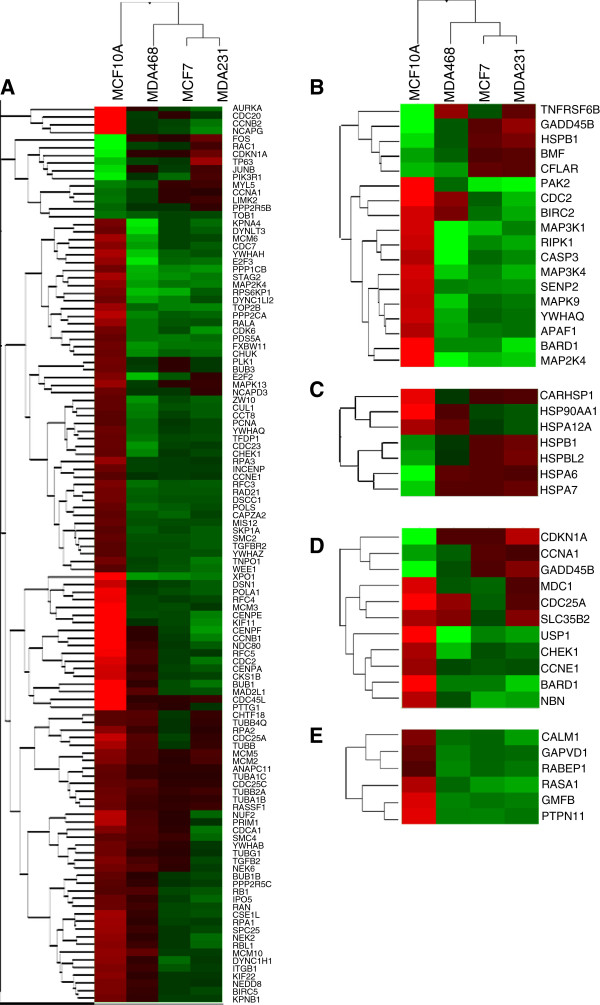
**Mammary epithelial cells respond to fever range hyperthermia through transcriptional alterations in gene networks unique from that of breast cancer cells.** Hierarchical clustered heatmaps depicting the transcriptional expression changes for genes involved in cell cycle **(A)**, apoptosis **(B)**, heatshock **(C)**, DNA damage **(D)**, and Ran/Rab **(E)** regulation in the *C* vs *H* and *C’* vs *H’* comparison (*red?=?overexpressed, green?=?underexpressed*).

Hierarchical clustering of the gene expression changes based on each cell line indicates that the breast cancer lines responded to hyperthermia more similarly to each other than to the mammary epithelial line (Figure 1A). Using a Venn diagram that strictly eliminated any genes with less than a 2-fold expression change (p < 0.05), we compared the gene expression profiles that were shared and unique between each cell line in response to hyperthermia, revealing that while many gene expression changes were common between one or more of the breast cancer lines, not a single 2-fold or greater gene expression change was shared between the mammary epithelial line and all three breast cancer lines (Figure 1B). This data strongly suggested that the hyperthermic response of breast cancer cells is truly distinct from that of mammary epithelial cells. As an independent assessment, we generated profile plots depicting the changes in normalized intensity values between the four cell lines, revealing that many of the statistically significant gene expression alterations we identified were largely shared between the three breast cancer lines and distinctly unique from that of the MCF10A line (Figure 1C). Using Metacore network analysis of the microarray data, we identified key signaling pathways that were unique to the mammary epithelial line and the three breast cancer lines. The hyperthermic response of MFC10A was strongly indicative of statistically significant gene expression alterations in a large number of genes involved in cell cycle regulation, apoptosis, heat shock response, and DNA damage response, (Figure 2A-D, Table 2) and changes in the expression of genes involved in these biological pathways were not observed in the three breast cancer lines. Network analysis indicated that signaling pathways with the highest statistical significance amongst the three breast cancer lines responding to hyperthermia (but not in the MCF10A line) included genes involved in Ras and Rab5A G-protein regulation (*GAPVD1, RASA1, RABEP1, CALM1, GMFB, PTPN11*) (Figure 2E, Table 2) and survival/apoptosis (*MAP2K4, BIRC2*) (Figure 2B, Table 2).

**Table 2:** **List of genes involved in the gene networks that are differentially regulated between mammary epithelial cells in the (*C *** **vs *H and C’ *** **vs *H’*) comparison**

**Gene symbol**	**Gene name**	**Accession number**	**MCF-10A**	**MCF-7**	**MDA-231**	**MDA-468**
DNA Damage						
USP1	Ubiquitin specific peptidase 1, TV3	NM_001017416.1	4.8	-1.8	-2.2	-5.3
CDC25A	Cell division cycle 25A, TV1	NM_001789.2	4.6	-1.3	1.2	2.0
BARD1	BRCA1 associated RING domain 1	NM_000465.1	3.5	-1.7	-2.8	-1.7
MDC1	Mediator of DNA-damage checkpoint 1	NM_014641.1	3.0	-1.4	1.1	-1.2
NBN	Nibrin	NM_002485.4	2.5	-2.4	-2.2	-1.1
SLC35B2	Solute carrier family 35, member B2	NM_178148.1	2.3	-1.0	1.8	1.8
CHEK1	Checkpoint kinase 1, TV3	NM_001274.3	2.3	-1.1	-1.4	-2.6
CCNE1	Cyclin E1, TV2	NM_057182.1	2.1	-1.2	-1.1	-1.0
CCNA1	Cyclin A1, TV1	NM_003914.2	-2.2	1.2	1.0	-1.3
GADD45B	Growth arrest and DNA-damage-inducible, beta	NM_015675.2	-3.8	1.1	1.8	-1.1
CDKN1A	Cyclin-dependent kinase inhibitor 1A (p21, Cip1), TV1	NM_000389.2	-4.7	1.1	2.5	1.1
Cell Cycle						
AURKA	Aurora kinase A, TV5	NM_198436.1	22.0	-1.3	-2.1	1.2
CDC20	Cell division cycle 20	NM_001255.2	18.0	1.1	-1.1	-1.2
CCNB2	Cyclin B2	NM_004701.2	17.5	-1.3	-2.3	-1.1
NCAPG	Non-SMC condensin I complex, subunit G, TV1	NM_022346.3	14.9	-1.4	-2.6	-1.1
BUB1	Budding uninhibited by benzimidazoles 1 homolog (yeast)	NM_004336.2	10.8	-1.5	-2.3	1.2
MAD2L1	MAD2 mitotic arrest deficient-like 1 (yeast)	NM_002358.2	9.8	-1.5	-1.6	2.1
CDC45L	Cell division cycle 45, TV2	NM_003504.3	8.0	1.1	1.4	1.1
CENPF	Centromere protein F, 350/400 kDa	NM_016343.3	7.8	-1.3	-3.0	1.1
PTTG1	Pituitary tumor-transforming 1	NM_004219.2	7.2	1.0	-1.0	1.4
MCM3	Minichromosome maintenance complex component 3, TV1	NM_002388.3	7.2	-1.5	-1.1	-1.4
CCNB1	Cyclin B1	NM_031966.2	6.9	-1.2	-2.5	1.1
CENPE	Centromere protein E, 312 kDa	NM_001813.2	6.6	-1.4	-2.5	-1.5
KIF11	Kinesin family member 11	NM_004523.2	6.5	-1.4	-2.7	-1.0
NDC80	NDC80 kinetochore complex component	NM_006101.1	6.5	-1.1	-1.8	1.4
XPO1	Exportin 1 (CRM1 homolog, yeast)	NM_003400.3	6.1	-2.7	-2.4	-3.0
RFC5	Replication factor C (activator 1) 5, 36.5 kDa, TV1	NM_007370.3	5.8	-1.2	-1.1	1.7
POLA1	Polymerase (DNA directed), alpha 1, catalytic subunit	NM_016937.2	5.7	-1.5	-1.8	-1.2
RFC4	Replication factor C (activator 1) 4, 37 kDa, TV2	NM_181573.1	5.6	-1.2	-1.4	-1.4
CDC2	Cyclin-dependent kinase 1, TV1	NM_001786.2	5.2	-1.2	-2.3	1.7
CENPA	Centromere protein A, TV2	NM_001042426.1	4.9	-1.3	-1.7	1.3
CKS1B	CDC28 protein kinase regulatory subunit 1B, TV1	NM_001826.1	4.8	-1.2	-1.3	1.6
DSN1	MIND kinetochore complex component, TV3	NM_024918.2	4.7	-1.3	-1.8	-1.1
CDC25A	Cell division cycle 25A, TV1	NM_001789.2	4.6	-1.3	1.2	2.0
NUF2	NDC80 kinetochore complex component, TV2	NM_031423.3	4.3	1.0	-2.0	1.7
PPP2CA	Protein phosphatase 2, catalytic subunit, alpha isozyme	NM_002715.2	4.3	-1.8	-2.0	-2.6
PRIM1	Primase, DNA, polypeptide 1 (49 kDa)	NM_000946.2	4.2	1.0	-1.0	1.8
YWHAH	Tyrosine 3-monooxygenase/tryptophan 5-monooxygenase activation protein, eta polypeptide	NM_003405.3	4.0	-1.9	-1.6	-3.9
TUBB	Tubulin, beta class I	NM_178014.2	3.9	-1.1	1.3	1.9
STAG2	Stromal antigen 2, TV2	NM_001042750.1	3.9	-2.8	-2.4	-3.4
CSE1L	CSE1 chromosome segregation 1-like (yeast), TV2	NM_177436.1	3.8	-1.4	-1.6	1.3
MCM6	Minichromosome maintenance complex component 6	NM_005915.4	3.8	-1.3	-1.4	-3.4
RPA1	Replication protein A1, 70 kDa	NM_002945.2	3.7	-1.3	-1.2	1.1
TOP2B	Topoisomerase (DNA) II beta 180 kDa	NM_001068.2	3.7	-2.5	-2.6	-2.0
RALA	V-ral simian leukemia viral oncogene homolog A (ras related)	NM_005402.2	3.6	-1.7	-1.6	-2.3
SPC25	SPC25, NDC80 kinetochore complex component	NM_020675.3	3.6	-1.1	-1.4	1.4
MAPK13	Mitogen-activated protein kinase 13, TV1	NM_002754.3	3.6	1.1	1.3	-1.2
RPA3	Replication protein A3, 14 kDa	NM_002947.3	3.5	-1.2	-1.1	-1.3
E2F3	E2F transcription factor 3, TV1	NM_001949.2	3.5	-2.0	-2.2	-4.5
MCM10	Minichromosome maintenance complex component 10, TV2	NM_018518.3	3.4	-1.2	-1.2	2.3
NEK2	NIMA-related kinase 2, TV1	NM_002497.2	3.4	-1.1	-2.1	1.1
MAP2K4	Mitogen-activated protein kinase kinase 4	NM_003010.2	3.3	-2.3	-2.5	-3.1
DYNC1H1	Dynein, cytoplasmic 1, heavy chain 1	NM_001376.2	3.3	-2.1	-1.1	1.2
KIF22	Kinesin family member 22, TV1	NM_007317.1	3.2	-1.1	-1.1	1.1
RPA2	Replication protein A2, 32 kDa	NM_002946.3	3.2	-1.0	1.0	1.4
CDC7	Cell division cycle 7, TV1	NM_003503.2	3.2	-1.3	-1.8	-3.1
RBL1	Retinoblastoma-like 1 (p107), TV1	NM_002895.2	3.2	-1.1	-2.0	1.4
MCM5	Minichromosome maintenance complex component 5	NM_006739.3	3.1	1.1	1.8	1.8
TGFB2	Transforming growth factor, beta 2	NM_003238.1	3.0	1.2	-1.4	1.7
E2F2	E2F transcription factor 2	NM_004091.2	3.0	-1.7	1.2	-3.7
CDCA1	Cell division cycle associated 1, TV1	NM_145697.1	3.0	1.1	-2.3	1.9
ITGB1	Integrin, beta 1 (fibronectin receptor, beta polypeptide, antigen CD29 includes MDF2, MSK12), TV1	NM_002211.2	3.0	-1.7	-1.2	1.4
MCM2	Minichromosome maintenance complex component 2, TV1	NM_004526.2	3.0	1.0	1.7	1.8
RFC3	Replication factor C (activator 1) 3, 38 kDa, TV1	NM_002915.3	2.9	-1.7	-1.8	-2.2
NEDD8	Neural precursor cell expressed, developmentally down-regulated 8	NM_006156.2	2.9	-1.1	-1.0	1.4
BIRC5	Baculoviral IAP repeat containing 5, TV3	NM_001012271.1	2.9	-1.1	-1.4	1.2
RPS6KB1	Ribosomal protein S6 kinase, 70 kDa, polypeptide 1, TV1	NM_003161.2	2.9	-3.4	-1.8	-1.2
KPNA4	Karyopherin alpha 4 (importin alpha 3)	NM_002268.3	2.8	-1.3	-2.1	-1.1
PPP1CB	Protein phosphatase 1, catalytic subunit, beta isozyme, TV3	NM_206876.1	2.7	-2.6	-2.9	-1.1
PLK1	Polo-like kinase 1	NM_005030.3	2.7	1.1	-1.1	1.2
CDK6	Cyclin-dependent kinase 6, TV1	NM_001259.5	2.7	-1.5	-2.9	2.1
NEK6	NIMA-related kinase 6, TV2	NM_014397.3	2.7	1.1	-1.2	1.1
KPNB1	Karyopherin (importin) beta 1, TV1	NM_002265.4	2.7	-1.2	-1.2	1.1
RAD21	RAD21 homolog (S. pombe)	NM_006265.1	2.7	-1.7	-1.7	1.4
DSCC1	Defective in sister chromatid cohesion 1	NM_024094.1	2.7	-1.6	-1.6	-1.4
TNPO1	Transportin 1, TV2	NM_153188.2	2.7	-1.6	-2.4	1.1
YWHAB	Tyrosine 3-monooxygenase/tryptophan 5-monooxygenase activation protein, beta polypeptide, TV1	NM_003404.3	2.6	1.1	-1.1	-1.5
TUBG1	Tubulin, gamma 1	NM_001070.3	2.6	1.0	-1.0	-1.0
FBXW11	F-box and WD repeat domain containing 11, TV1	NM_033645.2	2.6	-2.3	-2.3	1.4
BUB3	Budding uninhibited by benzimidazoles 3 homolog (yeast), TV2	NM_001007793.1	2.6	1.1	-1.1	-3.0
TUBA1B	Tubulin, alpha 1b	NM_006082.2	2.6	1.1	1.2	1.7
DYNC1LI2	Dynein, cytoplasmic 1, light intermediate chain 2	NM_006141.2	2.6	-2.5	-1.5	-1.2
TUBB2A	Tubulin, beta 2A class IIa	NM_001069.2	2.5	1.3	1.5	-1.4
CHUK	Conserved helix-loop-helix ubiquitous kinase	NM_001278.3	2.5	-1.9	-2.1	1.7
IPO5	Importin 5	NM_002271.4	2.5	-1.2	-2.0	1.3
RAN	RAN, member RAS oncogene family	NM_006325.2	2.5	-1.4	-1.5	1.6
RASSF1	Ras association (RalGDS/AF-6) domain family member 1, TVA	NM_007182.4	2.5	1.2	1.4	-1.1
INCENP	Inner centromere protein antigens 135/155 kDa, TV1	NM_001040694.1	2.5	-1.2	-1.2	2.0
DYNLT3	Dynein, light chain, Tctex-type 3	NM_006520.1	2.5	-1.3	-1.8	1.7
NCAPD3	Non-SMC condensin II complex, subunit D3	NM_015261.2	2.5	-1.1	1.2	-2.6
YWHAQ	Tyrosine 3-monooxygenase/tryptophan 5-monooxygenase activation protein, theta polypeptide	NM_006826.2	2.5	-1.2	-1.4	1.8
TFDP1	Transcription factor Dp-1, TV1	NM_007111.3	2.4	-1.4	-1.5	-3.9
PDS5A	PDS5, regulator of cohesion maintenance, TV2	NM_015200.1	2.4	-2.4	-2.1	1.9
MIS12	MIS12, MIND kinetochore complex component, TV2	NM_024039.1	2.4	-1.5	-1.5	-3.4
CDC23	Cell division cycle 23	NM_004661.3	2.4	-1.3	-1.2	1.3
POLS	PAP associated domain containing 7, TV1	NM_006999.3	2.4	-1.8	-1.5	-3.4
CDC25C	Cell division cycle 25C, TV1	NM_001790.3	2.3	1.1	1.1	1.1
BUB1B	BUB1 mitotic checkpoint serine/threonine kinase B	NM_001211.4	2.3	-1.1	-1.4	-2.0
ANAPC11	Anaphase promoting complex subunit 11, TV4	NM_001002246.1	2.3	1.1	1.1	-2.3
CHEK1	Checkpoint kinase 1, TV3	NM_001274.3	2.3	-1.1	-1.4	1.4
ZW10	Zw10 kinetochore protein	NM_004724.2	2.3	-1.4	-1.9	-1.2
SMC2	Structural maintenance of chromosomes 2, TV1	NM_001042550.1	2.3	-1.6	-1.8	-1.3
CUL1	Cullin 1	NM_003592.2	2.3	-1.6	-1.8	-4.5
TUBA1C	Tubulin, alpha 1c	NM_032704.3	2.2	1.0	1.0	2.3
SMC4	Structural maintenance of chromosomes 4, TV2	NM_001002800.1	2.2	1.4	-2.0	1.1
CAPZA2	Capping protein (actin filament) muscle Z-line, alpha 2	NM_006136.2	2.2	-2.0	-1.3	-3.1
PCNA	Proliferating cell nuclear antigen	NM_182649.1	2.1	-1.1	-1.4	1.2
PPP2R5C	Protein phosphatase 2, regulatory subunit B', gamma isoform , TV4	NM_178588.1	2.1	-1.0	-1.2	1.1
SKP1A	S-phase kinase-associated protein 1, TV1	NM_006930.2	2.1	-1.4	-1.8	1.4
TGFBR2	Transforming growth factor, beta receptor II (70/80 kDa), TV1	NM_001024847.1	2.1	-1.5	-1.8	-3.1
CHTF18	CTF18, chromosome transmission fidelity factor 18	NM_022092.1	2.1	-1.1	1.3	1.4
CCNE1	Cyclin E1, TV2	NM_057182.1	2.1	-1.2	-1.1	1.8
YWHAZ	Tyrosine 3-monooxygenase/tryptophan 5-monooxygenase activation protein, zeta polypeptide	NM_003406.2	2.1	-1.6	-1.6	1.7
CCT8	Chaperonin containing TCP1, subunit 8 (theta)	NM_006585.2	2.1	-1.3	-1.8	-3.7
TUBB4Q	Tubulin, beta polypeptide 4, member Q, pseudogene	NM_020040.3	2.1	-1.0	1.2	1.9
WEE1	WEE1 homolog (S. pombe), TV1	NM_003390.2	2.1	-1.7	-2.2	1.4
RB1	Retinoblastoma 1	NM_000321.2	2.0	-1.4	-1.7	1.8
PPP2R5B	Protein phosphatase 2, regulatory subunit B', beta	NM_006244.2	-2.0	-1.0	1.2	-2.2
CCNA1	Cyclin A1, TV1	NM_003914.2	-2.2	1.2	1.0	1.4
TOB1	Transducer of ERBB2, 1, TV1	NM_005749.2	-2.3	-1.3	-1.4	1.2
LIMK2	LIM domain kinase 2, TV1	NM_001031801.1	-2.3	1.2	1.5	-1.2
MYL5	Myosin, light chain 5, regulatory	NM_002477.1	-2.5	1.3	1.5	-1.1
TP63	Tumor protein p63, TV5	NM_001114981.1	-3.8	-1.1	3.7	-1.1
JUNB	Jun B proto-oncogene	NM_002229.2	-4.3	-1.2	1.6	1.2
PIK3R1	Phosphoinositide-3-kinase, regulatory subunit 1 (alpha), TV1	NM_181523.1	-4.7	-2.1	1.7	2.1
CDKN1A	Cyclin-dependent kinase inhibitor 1A (p21, Cip1)	NM_000389.2	-4.7	1.1	2.5	1.1
RAC1	Ras-related C3 botulinum toxin substrate 1 (rho family, small GTP binding protein Rac1), Rac1b	NM_018890.2	-7.6	-1.0	1.6	1.1
FOS	FBJ murine osteosarcoma viral oncogene homolog	NM_005252.2	-14.9	1.0	2.4	1.4
Apoptosis						
PAK2	p21 protein (Cdc42/Rac)-activated kinase 2	NM_002577.3	6.0	-2.9	-3.1	-1.3
CDC2	Cyclin-dependent kinase 1, TV1	NM_001786.2	5.2	-1.2	-2.3	1.7
BARD1	BRCA1 associated RING domain 1	NM_000465.1	3.5	-1.7	-2.8	-1.7
MAP2K4	Mitogen-activated protein kinase kinase 4	NM_003010.2	3.3	-2.3	-2.5	-3.1
MAP3K4	Mitogen-activated protein kinase kinase kinase 4, TV1	NM_005922.2	2.6	-1.6	-2.2	-2.5
MAP3K1	Mitogen-activated protein kinase kinase kinase 1, E3 ubiquitin protein ligase	NM_005921.1	2.6	-2.4	-1.6	-3.8
BIRC2	Baculoviral IAP repeat containing 2, TV1	NM_001166.3	2.6	-1.4	-2.1	1.5
SENP2	SUMO1/sentrin/SMT3 specific peptidase 2	NM_021627.2	2.5	-1.7	-1.8	-1.6
RIPK1	Receptor (TNFRSF)-interacting serine-threonine kinase 1	NM_003804.3	2.5	-1.7	-2.1	-4.5
YWHAQ	Tyrosine 3-monooxygenase/tryptophan 5-monooxygenase activation protein, theta polypeptide	NM_006826.2	2.5	-1.2	-1.4	-2.1
MAPK9	Mitogen-activated protein kinase 9, TV JNK2-a2	NM_002752.3	2.4	-1.3	-1.5	-2.5
CASP3	Caspase 3, apoptosis-related cysteine peptidase, TV beta	NM_032991.2	2.2	-1.3	-1.7	-4.0
APAF1	Apoptotic peptidase activating factor 1, TV1	NM_013229.2	2.2	-1.5	-1.4	-2.0
BMF	Bcl2 modifying factor, TV2	NM_033503.3	-2.0	1.0	1.1	-1.2
CFLAR	CASP8 and FADD-like apoptosis regulator	NM_003879.3	-2.3	1.2	1.1	-1.9
HSPB1	Heat shock 27 kDa protein 1	NM_001540.2	-2.7	1.2	1.5	-1.1
GADD45B	Growth arrest and DNA-damage-inducible, beta	NM_015675.2	-3.8	1.1	1.8	-1.1
TNFRSF6B	Tumor necrosis factor receptor superfamily, member 6b, decoy, transcript variant M68C	NM_032945.2	-4.3	-1.0	1.2	1.8
Heat Shock						
HSP90AA1	Heat shock protein 90 kDa, class A member 1	NM_001017963.2	4.0	-1.4	-1.7	1.1
CARHSP1	Calcium regulated heat stable protein 1, 24 kDa	NM_001042476.1	3.2	1.0	1.1	-1.1
HSPA12A	Heat shock protein 70 kDa 12A	NM_025015.2	2.3	-1.2	-1.7	1.4
HSPB1	Heat shock protein 27 kDa protein 1	NM_001540.2	-2.7	1.2	1.5	-1.1
HSPBL2	Heat shock 27 kDa protein 1 pseudogene 1	NR_024392.1	-3.2	1.2	1.6	-1.0
HSPA6	Heat shock 70 kDa protein 6	NM_002155.3	-5.3	1.2	1.1	1.3
HSPA7	Heat shock 70 kDa protein 7	NR_024151.1	-4.0	1.2	1.4	1.2

### Identification of hyperthermia induced genes that differentiate the heat shock response of mammary epithelial cells from that of breast cancer cells

Our previous analysis compared the hyperthermic response of each individual cell line to its transcriptional expression baseline at the normal growth temperature (*C* vs *H and C’* vs *H’*). Though this analysis provides us with information on how each individual cell line responds to hyperthermia relative to its normal growth temperature, it does not provide absolute comparisons of the transcriptome response of breast cancer cells relative to mammary epithelial cells following the elevated temperature. To better understand what provides breast cancer cells the selective disadvantage over mammary epithelial cells in response to hyperthermia we must identify those genes that are differentially expressed in breast cancer cell lines following hyperthermia from those of the mammary epithelial cell line following hyperthermia. To perform this analysis, we directly compared the gene expression changes that occurred for *H’* vs *H* and identified genes whose expression was truly distinct between the breast cancer and mammary epithelial cell lines following hyperthermia.

**Figure 3:**
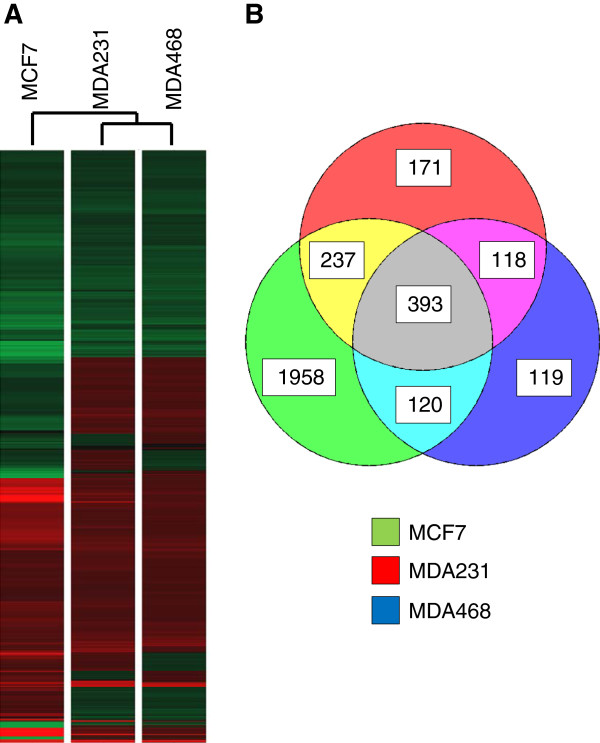
**Identification of genes that differentiate the transcriptional response of breast cancer cells following fever range hyperthermia. (A)** Heatmap depicting the two-fold or greater changes in gene expression (p < 0.05) occurring in at least one of the three breast cancer cell lines (MCF7, MDA231, MDA468) relative to the mammary epithelial cells in the *H’* vs *H* comparison (*red?=?overexpressed, green?=?underexpressed*). **(B)** Venn diagram illustrating common and unique 2-fold or greater gene expression changes (p < 0.05) between each of the breast cancer cell lines relative to the mammary epithelial cells in the *H’* vs *H* comparison.

When comparing *H’* (MCF7) vs *H* we identified 2708 genes whose expression was distinct at statistically significant levels (≥2 fold, p < 0.05). *H’* vs *H* comparisons of the MDA231 and MDA468 lines yielded 919 and 750 significant gene expression changes, respectively. Heatmap analysis indicated a strong trend in the gene expression profiles between each of the three breast cancer lines following hyperthermia (Figure 3A). Using a Venn diagram that strictly eliminated any genes with less than a 2-fold expression change (p < 0.05), we compared the gene expression alterations that were uniquely shared between all three cancer lines (*H’*) relative to the mammary epithelial line (*H*) (Figure 3B). This interpretation uncovered 393 genes whose 2-fold or greater changes in gene expression were differentially expressed in common amongst the three breast cancer lines following mild hyperthermic shock when compared to MCF10A cells following the same treatment (Table 3). These are the core genes that differentiate the hyperthermic response of breast cancer cells from that of mammary epithelial cells. In these data potentially lay the mechanism that may help define how mild hyperthermia preferentially selects against tumor cells.

**Table 3:** **List of genes that statistically distinguish the hyperthermic response of three breast cancer lines from the mammary epithelial cells in the *H’ *** **vs *H *** **analysis**

**Gene Symbol**	**Gene Name**	**Accession Number**	**MCF-7**	**MDA-231**	**MDA-468**
RN7SK	RNA, 7SK small nuclear	NR_001445.1	68.5	64.5	73.5
RN5S9	RNA, 5S ribosomal 9	NR_023371.1	54.5	64.7	64.9
RNU1-3	RNA, U1 small nuclear 3	NR_004408.1	44.2	27.9	30.3
RNU1G2	RNA, U1 small nuclear 4	NR_004426.1	42.5	26.4	28.5
KIAA1666	RIMS binding protein 3	XM_942124.2	38.5	36.1	41.6
RNU1-5	RNA, U1 small nuclear 5	NR_004400.1	36.5	25.1	28.1
SNORD3D	Small nucleolar RNA, C/D box 3D	NR_006882.1	34.8	43.3	47.2
RNU1A3	RNA, U1 small nuclear 1	NR_004430.1	30.2	19.9	20.3
SNORD3A	Small nucleolar RNA, C/D box 3A	NR_006880.1	30.1	42.6	43.8
SNORD3C	Small nucleolar RNA, C/D box 3C	NR_006881.1	28.4	30.9	34.7
RNY5	RNA, Ro-associated Y5	NR_001571.2	27.8	4.6	4.3
LOC100008589	RNA28S5 RNA, 28S ribosomal 5	NR_003287.1	27.2	28.9	30.9
LOC100132564	Hypothetical Protein LOC100132564	XM_001713808.1	26.7	26.5	27.3
LOC100132394	Hypothetical Protein	XP_001713861.1	23.8	23.1	22.9
RNU4-1	RNA, U4 small nuclear 1	NR_003925.1	18.5	11.6	13.1
RNU6ATAC	RNA, U6atac small nuclear (U12-dependent splicing)	NR_023344.1	16.3	11.8	12.4
LOC100134364	Hypothetical Protein LOC100134364	XM_001713810.1	15.9	15.5	17.2
HIST2H2AA3	Histone cluster 2, H2aa3	NM_003516.2	15.3	3.2	3.1
HIST2H2AA4	Histone cluster 2, H2aa4	NM_001040874.1	13.4	3.1	2.8
MIR1974	MicroRNA 1974	NR_031738.1	12.8	24.1	24.5
RMRP	RNA component of mitochondrial RNA processing endoribonuclease	NR_003051.2	12.1	14.1	15.6
RNA18S5	RNA18S5 RNA, 18S ribosomal 5	NR_003286.1	11.4	11.9	12.6
RNU4-2	RNA, U4 small nuclear 2	NR_003137.2	10.7	5.7	6.3
SCARNA20	Small Cajal body-specific RNA 20	NR_002999.2	7.7	3.2	3.1
LOC441763	Hypothetical Protein LOC441763	XM_930284.1	7.4	5.9	7.2
LOC728688	Ubiquitin-like, containing PHD and RING finger domains	XM_001724542.1	6.6	6.1	7.3
VTRNA1-1	Vault RNA 1-1	NR_026703.1	6.5	4.4	4.1
RNU6-1	RNA, U6 small nuclear 1	NR_004394.1	6.3	6.9	7.3
RNU6-15	RNA, U6 small nuclear 15	NR_028372.1	6.3	6.2	7.1
ALB	Albumin	NM_000477.3	4.9	5.3	5.5
HIST1H4H	Histone cluster 1, H4h	NM_003543.3	4.9	2.9	2.9
HIST2H4A	Histone cluster 2, H4a	NM_003548.2	4.6	5.1	4.7
TRK1	Transfer RNA lysine 1 (anticodon UUU)	NR_001449.1	4.6	3.5	3.8
SNORD46	Small nucleolar RNA, C/D box 46	NR_000024.2	4.2	3.9	3.8
RNU4ATAC	RNA, U4atac small nuclear (U12-dependent splicing)	NR_023343.1	4.2	3.7	3.9
KREMEN2	Kringle containing transmembrane protein 2	NM_145348.1	4.1	2.9	2.8
RNU11	RNA, U11 small nuclear	NR_004407.1	3.8	2.8	2.9
SNORA57	Small nucleolar RNA, H/ACA box 57	NR_004390.1	3.8	4.9	5.2
RPPH1	Ribonuclease P RNA component H1	NR_002312.1	3.7	2.7	3.1
SCARNA13	Small Cajal body-specific RNA 13	NR_003002.1	3.7	4.3	4.9
RPL12P6	Ribosomal protein L12 pseudogene 6	XR_016704.2	3.5	2.4	4.1
LOC389787	Tumor protein, translationally-controlled 1 pseudogene	XM_497072.2	3.4	3.1	2.1
RPL10L	Ribosomal protein L10-like	NM_080746.2	3.3	3.6	3.7
SNORA7B	Small nucleolar RNA, H/ACA box 7B	NR_002992.2	3.2	4.8	5.1
LOC100132673	Ribosomal protein S2 pseudogene 28	XR_039018.1	3.1	2.3	2.6
RNY4	RNA, Ro-associated Y4	NR_004393.1	3.1	5.5	4.7
HOXB6	Homeobox B6	NM_018952.4	2.9	4.2	4.1
HIST1H4K	Histone cluster 1, H4k	NM_003541.2	2.9	3.2	3.3
SNORA12	Small nucleolar RNA, H/ACA box 12	NR_002954.1	2.9	2.4	2.3
LOC643031	MT-ND5 pseudogene 10	XM_926402.1	2.8	2.9	3.8
HIST2H4B	Histone cluster 2, H4b	NM_001034077.4	2.7	2.8	2.9
HIST2H3D	Histone cluster 2, H3d	NM_001123375.1	2.3	3.7	3.9
HIST1H2AC	Histone cluster 1, H2ac	NM_003512.3	2.3	2.9	2.7
EGR1	Early growth response 1	NM_001964.2	2.3	2.8	2.4
SNORA63	Small nucleolar RNA, H/ACA box 63	NR_002586.1	2.3	7.8	7.3
SNORD13	Small nucleolar RNA, C/D box 13	NR_003041.1	2.3	5.6	5.7
RNY1	RNA, Ro-associated Y1	NR_004391.1	2.1	4.4	4.1
HIST1H2AM	Histone cluster 1, H2am	NM_003514.2	2.1	3.1	3.5
MARCH7	Membrane-associated ring finger 7, E3 ubiquitin protein ligase	NM_022826.2	-2.0	-2.3	-2.3
GPBP1	GC-rich promoter binding protein 1	NM_022913.1	-2.0	-3.4	-2.9
RAB11FIP1	RAB11 family interacting protein 1 (class I)	NM_001002814.1	-2.0	-2.0	-2.8
CEBPG	CCAAT/enhancer binding protein (C/EBP), gamma	NM_001806.2	-2.0	-2.0	-2.5
ZWILCH	Zwilch kinetochore protein	NR_003105.1	-2.0	-2.7	-2.6
DDX18	DEAD (Asp-Glu-Ala-Asp) box polypeptide 18	NM_006773.3	-2.0	-2.6	-2.7
PTP4A1	Protein tyrosine phosphatase type IVA, member 1	NM_003463.3	-2.0	-2.9	-2.4
DDX3X	DEAD (Asp-Glu-Ala-Asp) box polypeptide 3, X-linked	NM_001356.3	-2.0	-2.6	-2.3
CTCF	CCCTC-binding factor (zinc finger protein)	NM_006565.2	-2.0	-2.6	-2.3
SKAP2	src kinase associated phosphoprotein 2	NM_003930.3	-2.0	-2.3	-2.2
ANO6	Anoctamin 6	NM_001025356.1	-2.0	-2.3	-2.3
PPAT	Phosphoribosyl pyrophosphate amidotransferase	NM_002703.3	-2.0	-2.1	-2.3
USP34	Ubiquitin specific peptidase 34	NM_014709.3	-2.0	-2.1	-2.0
LHFPL2	Lipoma HMGIC fusion partner-like 2	NM_005779.1	-2.1	-2.1	-2.1
KIAA1147	KIAA1147	NM_001080392.1	-2.1	-2.1	-2.3
KIAA2010	SMEK homolog 1, suppressor of mek1 (Dictyostelium)	NM_032560.3	-2.1	-3.6	-3.0
DYRK1A	Dual-specificity tyrosine-(Y)-phosphorylation regulated kinase 1A	NM_130438.1	-2.1	-2.5	-2.3
RBBP8	Retinoblastoma binding protein 8	NM_203291.1	-2.1	-2.6	-2.4
ANKIB1	Ankyrin repeat and IBR domain containing 1	NM_019004.1	-2.1	-2.5	-2.5
C12orf32	RAD9-HUS1-RAD1 interacting nuclear orphan 1	NM_031465.2	-2.1	-2.1	-2.1
FNIP1	Folliculin interacting protein 1	NM_001008738.2	-2.1	-2.0	-2.1
NAE1	NEDD8 activating enzyme E1 subunit 1	NM_001018160.1	-2.2	-2.0	-2.3
RSBN1	Round spermatid basic protein 1	NM_018364.3	-2.2	-2.0	-2.3
SUPT16H	Suppressor of Ty 16 homolog (S. cerevisiae)	NM_007192.2	-2.2	-2.1	-2.2
TMED10	Transmembrane emp24-like trafficking protein 10 (yeast)	NM_006827.5	-2.2	-3.6	-2.9
PPP1CB	Protein phosphatase 1, catalytic subunit, beta isozyme	NM_206876.1	-2.2	-2.4	-2.7
ARL6IP1	ADP-ribosylation factor-like 6 interacting protein 1	NM_015161.1	-2.2	-2.8	-2.7
ASNSD1	Asparagine synthetase domain containing 1	NM_019048.1	-2.2	-2.9	-2.5
NRBF2P4	Nuclear receptor binding factor 2 pseudogene 4	XM_001127763.1	-2.2	-2.6	-2.4
TTC37	Tetratricopeptide repeat domain 37	NM_014639.2	-2.2	-2.6	-2.2
DICER1	Dicer 1, ribonuclease type III	NM_030621.2	-2.2	-2.6	-2.3
UBE2G1	Ubiquitin-conjugating enzyme E2G 1	NM_003342.4	-2.2	-2.5	-2.4
LRPPRC	Leucine-rich pentatricopeptide repeat containing	NM_133259.2	-2.2	-2.4	-2.2
OTUD4	OTU domain containing 4	NM_199324.1	-2.2	-2.3	-2.2
FUBP3	Far upstream element (FUSE) binding protein 3	NM_003934.1	-2.2	-2.3	-2.2
FAM175B	Family with sequence similarity 175, member B	NM_032182.3	-2.2	-2.1	-2.1
DDX50	DEAD (Asp-Glu-Ala-Asp) box polypeptide 50	NM_024045.1	-2.2	-2.0	-2.1
PIGA	Phosphatidylinositol glycan anchor biosynthesis, class A	NM_020473.2	-2.2	-2.0	-2.2
FAM168B	Family with sequence similarity 168, member B	NM_001009993.2	-2.3	-2.0	-2.0
FBXO11	F-box protein 11	NM_025133.3	-2.3	-2.1	-2.5
SLC25A24	Solute carrier family 25 (mitochondrial carrier; phosphate carrier), member 24	NM_013386.3	-2.3	-3.4	-2.5
NCKAP1	NCK-associated protein 1	NM_013436.3	-2.3	-3.3	-2.7
FBXW11	F-box and WD repeat domain containing 11	NM_033645.2	-2.3	-2.5	-2.7
SLC30A9	Solute carrier family 30 (zinc transporter), member 9	NM_006345.3	-2.3	-2.0	-2.7
ATAD2	ATPase family, AAA domain containing 2	NM_014109.2	-2.3	-2.8	-2.0
CSNK1A1	Casein kinase 1, alpha 1	NM_001025105.1	-2.3	-2.5	-2.2
CHUK	Conserved helix-loop-helix ubiquitous kinase	NM_001278.3	-2.3	-2.2	-2.3
ATP6V1C1	ATPase, H?+?transporting, lysosomal 42 kDa, V1 subunit C1	NM_001695.4	-2.3	-2.1	-2.4
CUL2	Cullin 2	NM_003591.2	-2.3	-2.2	-2.1
BRMS1L	Breast cancer metastasis-suppressor 1-like	NM_032352.3	-2.3	-2.1	-2.0
UBQLN1	Ubiquilin 1	NM_013438.3	-2.3	-2.1	-2.1
OPA1	Optic atrophy 1 (autosomal dominant)	NM_015560.1	-2.4	-2.7	-2.6
EFR3A	EFR3 homolog A (S. cerevisiae)	NM_015137.3	-2.4	-2.3	-3.2
BAT2D1	Proline-rich coiled-coil 2C	NM_015172.3	-2.4	-2.5	-2.1
OSBPL9	Oxysterol binding protein-like 9	NM_148906.1	-2.4	-2.6	-2.5
CDR2	Cerebellar degeneration-related protein 2, 62 kDa	NM_001802.1	-2.4	-2.3	-2.2
KPNA4	Karyopherin alpha 4 (importin alpha 3)	NM_002268.3	-2.4	-2.2	-2.4
ARL1	ADP-ribosylation factor-like 1	NM_001177.3	-2.4	-2.2	-2.3
DHX32	DEAH (Asp-Glu-Ala-His) box polypeptide 32	NM_018180.2	-2.4	-2.1	-2.3
RIOK3	RIO kinase 3	NM_003831.3	-2.4	-2.0	-2.1
C5orf51	Chromosome 5 open reading frame 51	NM_175921.4	-2.5	-2.8	-2.6
TJP1	Tight junction protein 1	NM_003257.3	-2.5	-2.4	-2.2
CSNK1G3	Casein kinase 1, gamma 3	NM_001031812.2	-2.5	-2.4	-2.3
PREI3	MOB family member 4, phocein	NM_199482.1	-2.5	-2.4	-2.4
KIAA0494	EF-hand calcium binding domain 14	NM_014774.1	-2.5	-2.3	-2.4
PRKRIR	Protein-kinase, interferon-inducible double stranded RNA dependent inhibitor, repressor of (P58 repressor)	NM_004705.2	-2.5	-2.7	-3.0
LOC644363	LOC644363	XR_016912.2	-2.5	-2.6	-2.9
DCP2	DCP2 decapping enzyme homolog (S. cerevisiae)	NM_152624.4	-2.5	-2.5	-3.0
ROD1	Polypyrimidine tract binding protein 3	NM_005156.4	-2.5	-2.9	-3.2
CRK	v-crk sarcoma virus CT10 oncogene homolog (avian)	NM_016823.2	-2.5	-3.3	-2.8
PRKAR1A	Protein kinase, cAMP-dependent, regulatory, type I, alpha	NM_002734.3	-2.5	-2.6	-2.4
GPSM2	G-protein signaling modulator 2	NM_013296.3	-2.5	-2.2	-2.3
API5	Apoptosis inhibitor 5	NM_006595.2	-2.5	-2.1	-2.5
CRKL	v-crk sarcoma virus CT10 oncogene homolog (avian)-like	NM_005207.2	-2.5	-2.0	-2.6
CBL	Cbl proto-oncogene, E3 ubiquitin protein ligase	NM_005188.2	-2.5	-2.0	-2.2
SMG1	smg-1 homolog, phosphatidylinositol 3-kinase-related kinase interferon	NM_015092.3	-2.6	-2.9	-2.6
IRF2BP2	Regulatory factor 2 binding protein 2	NM_182972.2	-2.6	-2.7	-2.7
CNOT6	CCR4-NOT transcription complex, subunit 6	NM_015455.3	-2.6	-2.7	-2.6
THUMPD1	THUMP domain containing 1	NM_017736.3	-2.6	-2.6	-2.5
BMI1	BMI1 polycomb ring finger oncogene	NM_005180.5	-2.6	-2.4	-2.7
CDC2L6	Cyclin-dependent kinase 19	NM_015076.3	-2.6	-2.4	-2.8
TULP4	Tubby like protein 4	NM_001007466.1	-2.6	-2.3	-2.6
LARP4B	La ribonucleoprotein domain family, member 4B	NM_015155.1	-2.6	-2.3	-2.3
HECTD1	HECT domain containing E3 ubiquitin protein ligase 1	NM_015382.1	-2.6	-2.6	-2.4
CPSF2	Cleavage and polyadenylation specific factor 2, 100 kDa	NM_017437.1	-2.6	-2.8	-2.6
PDS5A	PDS5, regulator of cohesion maintenance, homolog A (S. cerevisiae)	NM_015200.1	-2.6	-2.6	-2.9
RIPK1	Receptor (TNFRSF)-interacting serine-threonine kinase 1	NM_003804.3	-2.6	-2.1	-2.4
DCAF6	DDB1 and CUL4 associated factor 6	NM_001017977.1	-2.6	-2.1	-2.4
KIAA1429	KIAA1429	NM_015496.3	-2.6	-2.0	-2.3
RUNX1	Runt-related transcription factor 1	NM_001754.3	-2.6	-2.1	-2.2
CEP135	Centrosomal protein 135 kDa	NM_025009.3	-2.6	-2.0	-2.2
MBNL1	Muscleblind-like splicing regulator 1	NM_207296.1	-2.6	-2.5	-2.2
RBPJ	Recombination signal binding protein for immunoglobulin kappa J region	NM_203284.1	-2.6	-2.4	-2.0
USP16	Ubiquitin specific peptidase 16	NM_006447.2	-2.6	-2.2	-2.1
TOMM20	Translocase of outer mitochondrial membrane 20 homolog (yeast)	NM_014765.1	-2.7	-2.3	-2.5
MTX3	Metaxin 3	NM_001010891.3	-2.7	-2.7	-2.6
RAPH1	Ras association (RalGDS/AF-6) and pleckstrin homology domains 1	NM_213589.1	-2.7	-2.6	-2.5
PHF20L1	PHD finger protein 20-like 1	NM_016018.4	-2.7	-2.6	-2.7
UBP1	Upstream binding protein 1 (LBP-1a)	NM_014517.3	-2.7	-2.5	-2.6
GBE1	Glucan (1,4-alpha-), branching enzyme 1	NM_000158.2	-2.7	-2.4	-2.3
CUL4B	Cullin 4B	NM_001079872.1	-2.7	-2.4	-2.4
PAPOLA	Poly(A) polymerase alpha	NM_001037281.1	-2.7	-2.4	-2.9
RNMT	RNA (guanine-7-) methyltransferase	NM_003799.1	-2.7	-2.1	-2.4
FBXO34	F-box protein 34	NM_017943.2	-2.7	-2.3	-2.1
DOCK7	Dedicator of cytokinesis 7	NM_033407.2	-2.7	-2.3	-2.1
BTBD3	BTB (POZ) domain containing 3	NM_014962.2	-2.7	-2.2	-2.1
C6orf130	O-acyl-ADP-ribose deacylase 1	NM_145063.2	-2.8	-2.3	-2.1
MRPL35	Mitochondrial ribosomal protein L35	NM_016622.2	-2.8	-2.2	-2.2
PUM2	Pumilio homolog 2 (Drosophila)	NM_015317.1	-2.8	-2.8	-2.8
NUDT21	Nudix (nucleoside diphosphate linked moiety X)-type motif 21	NM_007006.2	-2.8	-2.7	-2.3
ICK	Intestinal cell (MAK-like) kinase	NM_016513.3	-2.8	-2.7	-2.5
RBM17	RNA binding motif protein 17	NM_032905.3	-2.8	-2.4	-2.1
RMI1	RMI1, RecQ mediated genome instability 1, homolog (S. cerevisiae)	NM_024945.2	-2.9	-2.4	-2.1
MAP2K4	Mitogen-activated protein kinase kinase 4	NM_003010.2	-2.9	-2.8	-2.8
G3BP2	GTPase activating protein (SH3 domain) binding protein 2	NM_203504.1	-2.9	-2.5	-2.5
NAMPT	Nicotinamide phosphoribosyltransferase	NM_005746.2	-2.9	-2.7	-2.1
BEND7	BEN domain containing 7	NM_001100912.1	-2.9	-2.2	-2.1
FEZ2	Fasciculation and elongation protein zeta 2 (zygin II)	NM_005102.2	-3.0	-2.2	-2.2
ARL4A	ADP-ribosylation factor-like 4A	NM_001037164.1	-3.0	-2.4	-2.2
SOCS4	Suppressor of cytokine signaling 4	NM_080867.2	-3.0	-3.0	-2.6
STAG2	Stromal antigen 2	NM_001042750.1	-3.0	-2.9	-2.5
C14orf32	Mitogen-activated protein kinase 1 interacting protein 1-like	NM_144578.2	-3.0	-2.9	-2.9
PPP3CB	Protein phosphatase 3, catalytic subunit, beta isozyme	NM_021132.1	-3.0	-2.4	-2.6
RBM25	RNA binding motif protein 25	NM_021239.1	-3.0	-2.4	-2.5
ERI1	Exoribonuclease 1	NM_153332.3	-3.0	-2.0	-2.3
NRD1	Nardilysin (N-arginine dibasic convertase)	NM_002525.1	-3.0	-2.4	-2.1
E2F3	E2F transcription factor 3	NM_001949.2	-3.0	-2.0	-2.0
ANKRD28	Ankyrin repeat domain 28	NM_015199.2	-3.1	-2.3	-2.2
ZAK	Sterile alpha motif and leucine zipper containing kinase AZK	NM_133646.2	-3.1	-3.1	-3.4
HNRPR	Heterogeneous nuclear ribonucleoprotein R	NM_005826.2	-3.1	-2.9	-2.1
TMEM123	Transmembrane protein 123	NM_052932.2	-3.1	-2.8	-2.3
FAM178A	Family with sequence similarity 178, member A	NM_018121.3	-3.1	-2.7	-2.6
EML4	Echinoderm microtubule associated protein like 4	NM_019063.2	-3.1	-2.6	-2.6
FOXJ3	Forkhead box J3	NM_014947.3	-3.1	-2.5	-2.5
NT5DC3	5'-nucleotidase domain containing 3	NM_016575.1	-3.1	-2.3	-2.6
LPP	LIM domain containing preferred translocation partner in lipoma	NM_005578.2	-3.1	-2.2	-3.5
RND3	Rho family GTPase 3	NM_005168.3	-3.1	-2.0	-2.2
WDR36	WD repeat domain 36	NM_139281.2	-3.1	-2.0	-2.2
CDCA1	NUF2, NDC80 kinetochore complex component	NM_145697.1	-3.1	-2.5	-2.0
PRSS23	Protease, serine, 23	NM_007173.4	-3.2	-2.3	-2.0
ERCC6L	Excision repair cross-complementing rodent repair deficiency, complementation group 6-like	NM_017669.2	-3.2	-2.3	-2.0
FAM122B	Family with sequence similarity 122B	NM_032448.1	-3.3	-2.5	-2.0
CKAP5	Cytoskeleton associated protein 5	NM_001008938.1	-3.3	-2.2	-2.1
CGGBP1	CGG triplet repeat binding protein 1	NM_001008390.1	-3.3	-2.8	-3.5
TBL1XR1	Transducin (beta)-like 1 X-linked receptor 1	NM_024665.3	-3.3	-3.1	-2.7
LOC644799	LOC644799	XM_934554.1	-3.3	-3.0	-2.5
DCK	Deoxycytidine kinase	NM_000788.1	-3.3	-2.8	-2.2
SERBP1	SERPINE1 mRNA binding protein 1	NM_030666.2	-3.3	-2.1	-3.3
PPP1CC	Protein phosphatase 1, catalytic subunit, gamma isozyme	NM_002710.1	-3.3	-2.2	-2.1
KIF5B	Kinesin family member 5B	NM_004521.1	-3.4	-4.1	-3.3
AFF4	AF4/FMR2 family, member 4	NM_014423.3	-3.4	-3.6	-3.5
MPP5	Membrane protein, palmitoylated 5 (MAGUK p55 subfamily member 5)	NM_022474.2	-3.4	-3.1	-2.7
IPO5	Importin 5	NM_002271.4	-3.4	-2.6	-2.2
HNRNPR	Heterogeneous nuclear ribonucleoprotein R	NM_005826.3	-3.4	-2.6	-2.3
CP110	Centriolar coiled coil protein 110 kDa	NM_014711.3	-3.4	-2.5	-2.5
FEM1C	fem-1 homolog c (C. elegans)	NM_020177.2	-3.4	-2.4	-2.5
PHTF1	Putative homeodomain transcription factor 1	NM_006608.1	-3.4	-2.3	-2.1
RAD51AP1	RAD51 associated protein 1	NM_006479.3	-3.4	-2.2	-2.0
MAPRE1	Microtubule-associated protein, RP/EB family, member 1	NM_012325.1	-3.4	-2.1	-2.1
TMPO	Thymopoietin	NM_003276.1	-3.5	-3.1	-2.3
LACTB	Lactamase, beta	NM_032857.2	-3.5	-2.3	-2.3
DDX46	DEAD (Asp-Glu-Ala-Asp) box polypeptide 46	NM_014829.2	-3.5	-2.3	-2.4
SPEN	Spen homolog, transcriptional regulator (Drosophila)	NM_015001.2	-3.5	-2.0	-2.7
TMEM19	Transmembrane protein 19	NM_018279.3	-3.5	-2.1	-2.0
CBFB	Core-binding factor, beta subunit	NM_001755.2	-3.5	-2.2	-2.2
IPO8	Importin 8	NM_006390.2	-3.5	-2.2	-2.1
WT1	Wilms tumor 1	NM_024426.3	-3.5	-2.4	-2.1
CKAP2	Cytoskeleton associated protein 2	NM_001098525.1	-3.6	-3.0	-2.4
WEE1	WEE1 homolog (S. pombe)	NM_003390.2	-3.6	-2.8	-2.7
PDCD6IP	Programmed cell death 6 interacting protein	NM_013374.3	-3.6	-2.7	-2.3
ZNF788	Zinc finger family member 788	XR_041527.1	-3.6	-2.6	-2.3
RAP2A	RAP2A, member of RAS oncogene family	NM_021033.5	-3.6	-2.3	-2.7
MGEA5	Meningioma expressed antigen 5 (hyaluronidase)	NM_012215.2	-3.6	-2.1	-2.7
UBE3A	Ubiquitin protein ligase E3A	NM_000462.2	-3.6	-2.0	-2.8
PLK4	Polo-like kinase 4	NM_014264.3	-3.7	-3.8	-3.2
RP2	Retinitis pigmentosa 2 (X-linked recessive)	NM_006915.1	-3.7	-2.9	-2.5
SETD2	SET domain containing 2	NM_014159.4	-3.7	-2.7	-2.8
KLHL5	Kelch-like family member 5	NM_001007075.1	-3.7	-2.4	-2.6
KBTBD2	Kelch repeat and BTB (POZ) domain containing 2	NM_015483.1	-3.7	-3.7	-3.1
USP9X	Ubiquitin specific peptidase 9, X-linked	NM_001039591.2	-3.8	-2.8	-2.4
RAB23	RAB23, member RAS oncogene family	NM_016277.3	-3.8	-2.6	-2.5
DR1	Down-regulator of transcription 1, TBP-binding (negative cofactor 2)	NM_001938.2	-3.8	-2.3	-2.2
RAB8B	RAB8B, member RAS oncogene family	NM_016530.2	-3.8	-2.3	-2.5
ZNF451	Zinc finger protein 451	NM_001031623.2	-3.8	-2.2	-2.4
ZZZ3	Zinc finger, ZZ-type containing 3	NM_015534.4	-3.9	-3.1	-3.3
CDC2	Cyclin-dependent kinase 1	NM_001786.2	-4.0	-2.9	-2.3
ZFP106	Zinc finger protein 106	NM_022473.1	-4.0	-3.0	-2.5
CAB39	Calcium binding protein 39	NM_016289.2	-4.0	-2.0	-3.1
TNPO1	Transportin 1	NM_153188.2	-4.0	-2.9	-2.5
MAP3K4	Mitogen-activated protein kinase kinase kinase 4	NM_005922.2	-4.1	-2.3	-2.7
ECT2	Epithelial cell transforming sequence 2 oncogene	NM_018098.4	-4.1	-2.3	-2.0
TMED5	Transmembrane emp24 protein transport domain containing 5	NM_016040.3	-4.1	-2.3	-2.1
SEH1L	SEH1-like (S. cerevisiae)	NM_001013437.1	-4.1	-2.7	-2.5
NCAPG2	Non-SMC condensin II complex, subunit G2	NM_017760.5	-4.2	-2.7	-2.2
USP1	Ubiquitin specific peptidase 1	NM_001017416.1	-4.2	-2.3	-2.4
OXSR1	Oxidative-stress responsive 1	NM_005109.2	-4.2	-2.3	-2.4
PTPN12	Protein tyrosine phosphatase, non-receptor type 12	NM_002835.2	-4.2	-2.8	-2.8
CMPK1	Cytidine monophosphate (UMP-CMP) kinase 1, cytosolic	NM_016308.1	-4.2	-2.5	-2.4
PAFAH1B1	Platelet-activating factor acetylhydrolase 1b, regulatory subunit 1 (45 kDa)	NM_000430.2	-4.3	-3.8	-3.1
PURB	Purine-rich element binding protein B	NM_033224.3	-4.3	-2.5	-3.5
STK4	Serine/threonine kinase 4	NM_006282.2	-4.3	-2.3	-3.3
KATNAL1	Katanin p60 subunit A-like 1	NM_001014380.1	-4.3	-3.1	-2.9
NIN	Ninein (GSK3B interacting protein)	NM_020921.3	-4.3	-2.7	-2.4
LOC283267	Long intergenic non-protein coding RNA 294	NR_015451.1	-4.3	-2.6	-2.6
CCNB1	Cyclin B1	NM_031966.2	-4.3	-2.4	-2.2
YAP1	Yes-associated protein 1	NM_006106.2	-4.3	-2.0	-2.5
XPO1	Exportin 1 (CRM1 homolog, yeast)	NM_003400.3	-4.4	-2.9	-2.6
PTPN11	Protein tyrosine phosphatase, non-receptor type 11	NM_002834.3	-4.4	-2.4	-2.5
PHF3	PHD finger protein 3	NM_015153.1	-4.4	-2.2	-2.6
VMA21	Vacuolar H?+?-ATPase homolog (S. cerevisiae)	NM_001017980.2	-4.4	-2.2	-2.3
CHST15	Carbohydrate sulfotransferase 15	NM_015892.2	-4.4	-2.0	-2.5
RUNX2	Runt-related transcription factor 2	NM_001024630.2	-4.5	-2.7	-2.4
KIF14	Kinesin family member 14	NM_014875.1	-4.6	-3.4	-2.2
EHBP1	EH domain binding protein 1	NM_015252.2	-4.6	-2.7	-2.6
NEK2	NIMA-related kinase 2	NM_002497.2	-4.6	-2.7	-2.6
STK38	Serine/threonine kinase 38	NM_007271.2	-4.7	-3.2	-2.4
ZNF22	Zinc finger protein 22	NM_006963.3	-4.7	-3.2	-2.8
SPRY4	Sprouty homolog 4 (Drosophila)	NM_030964.2	-4.7	-2.0	-2.1
GMFB	Glia maturation factor, beta	NM_004124.2	-4.8	-2.2	-3.2
GCNT1	Glucosaminyl (N-acetyl) transferase 1, core 2	NM_001097635.1	-4.8	-3.1	-2.4
HERC4	HECT and RLD domain containing E3 ubiquitin protein ligase 4	NM_022079.2	-4.8	-3.1	-2.6
PPP4R1	Protein phosphatase 4, regulatory subunit 1	NM_005134.2	-4.8	-2.1	-2.6
SMAD5	SMAD family member 5	NM_005903.5	-4.9	-2.2	-3.2
GNG12	guanine nucleotide binding protein (G protein), gamma 12	NM_018841.4	-4.9	-2.8	-2.1
SMARCA1	SWI/SNF related, matrix associated, actin dependent regulator of chromatin, subfamily a, member 1	NM_003069.2	-4.9	-2.8	-2.3
DEK	DEK oncogene	NM_003472.2	-4.9	-2.5	-2.4
FAM107B	Family with sequence similarity 107, member B	NM_031453.2	-5.1	-3.1	-2.1
SUZ12	Suppressor of zeste 12 homolog (Drosophila)	NM_015355.1	-5.1	-2.8	-2.7
OSBPL3	Oxysterol binding protein-like 3	NM_145322.1	-5.1	-2.7	-2.7
UBE3C	Ubiquitin protein ligase E3C	NM_014671.1	-5.1	-2.7	-2.7
HSDL2	Hydroxysteroid dehydrogenase like 2	NM_032303.3	-5.1	-2.5	-2.5
C14orf106	MIS18 binding protein 1	NM_018353.3	-5.1	-2.3	-2.3
MBP	Myelin basic protein	NM_001025100.1	-5.1	-2.0	-2.3
GPAM	Glycerol-3-phosphate acyltransferase, mitochondrial	NM_020918.3	-5.2	-3.1	-2.7
RASA1	RAS p21 protein activator (GTPase activating protein) 1	NM_002890.1	-5.2	-2.9	-3.1
KIF11	Kinesin family member 11	NM_004523.2	-5.2	-3.3	-3.1
FBXO5	F-box protein 5	NM_012177.2	-5.2	-3.1	-2.3
CENPE	Centromere protein E, 312 kDa	NM_001813.2	-5.2	-2.6	-2.3
PAK2	p21 protein (Cdc42/Rac)-activated kinase 2	NM_002577.3	-5.3	-3.3	-3.6
IL7R	Interleukin 7 receptor	XM_937367.1	-5.3	-2.8	-2.2
ENC1	Ectodermal-neural cortex 1 (with BTB domain)	NM_003633.1	-5.3	-2.6	-2.3
SOX9	SRY (sex determining region Y)-box 9	NM_000346.2	-5.3	-2.5	-2.3
ASXL1	Additional sex combs like 1 (Drosophila) (ASXL1), TV1	NM_015338.4	-5.3	-2.0	-2.0
C10orf6	Family with sequence similarity 178, member A	NM_018121.2	-5.5	-3.2	-3.4
CEP55	Centrosomal protein 55 kDa	NM_018131.3	-5.6	-3.4	-2.6
NMT2	N-myristoyltransferase 2	NM_004808.2	-5.6	-2.4	-2.0
PPPDE1	Desumoylating isopeptidase 2	NM_016076.3	-5.8	-2.4	-2.9
TGFBR2	Transforming growth factor, beta receptor II (70/80 kDa)	NM_001024847.1	-6.0	-2.1	-2.2
MID1	Midline 1 (Opitz/BBB syndrome)	NM_033290.2	-6.2	-2.9	-2.1
FNDC3B	Fibronectin type III domain containing 3B	NM_001135095.1	-6.2	-2.8	-2.6
BIRC2	Baculoviral IAP repeat containing 2	NM_001166.3	-6.2	-2.9	-2.7
FAM3C	Family with sequence similarity 3, member C	NM_001040020.1	-6.2	-2.4	-2.0
KIF23	Kinesin family member 23	NM_004856.4	-6.3	-3.4	-2.7
CLIC4	Chloride intracellular channel 4	NM_013943.1	-6.3	-3.4	-2.6
PDGFC	Platelet derived growth factor C	NM_016205.1	-6.4	-2.4	-2.0
PRKCA	Protein kinase C, alpha	NM_002737.2	-6.7	-2.5	-2.2
NCAPG	Non-SMC condensin I complex, subunit G	NM_022346.3	-7.1	-2.6	-2.1
CENPF	Centromere protein F, 350/400 kDa	NM_016343.3	-7.4	-2.9	-2.7
GCNT2	Glucosaminyl (N-acetyl) transferase 2, I-branching enzyme (I blood group)	NM_001491.2	-7.4	-2.5	-2.5
C14orf135	Pecanex-like 4 (Drosophila)	NM_022495.5	-7.5	-2.7	-2.5
PBK	PDZ binding kinase	NM_018492.2	-7.8	-3.3	-2.4
TOP2A	Topoisomerase (DNA) II alpha 170 kDa	NM_001067.2	-8.1	-3.8	-2.7
CAV2	Caveolin 2	NM_001233.3	-8.1	-2.3	-2.5
SERTAD2	SERTA domain containing 2	NM_014755.1	-8.3	-3.1	-3.6
ACSL4	Acyl-CoA synthetase long-chain family member 4	NM_004458.1	-8.5	-2.5	-2.5
FAM83D	Family with sequence similarity 83, member D	NM_030919.2	-9.2	-4.3	-3.8
CDK6	Cyclin-dependent kinase 6	NM_001259.5	-9.4	-3.1	-4.1
FRMD6	FERM domain containing 6	NM_152330.3	-9.5	-3.1	-2.4
SNAPC1	Small nuclear RNA activating complex, polypeptide 1, 43 kDa	NM_003082.2	-9.8	-2.3	-2.0
CALD1	Caldesmon 1	NM_033140.2	-9.9	-2.1	-2.5
BCAT1	Branched chain amino-acid transaminase 1, cytosolic	NM_005504.4	-10.2	-5.6	-4.2
DLGAP5	Discs, large (Drosophila) homolog-associated protein 5	NM_014750.3	-10.4	-3.1	-2.4
ANLN	Anillin, actin binding protein	NM_018685.2	-10.9	-3.1	-2.2
TACC1	Transforming, acidic coiled-coil containing protein 1	NM_006283.1	-11.1	-2.7	-2.6
AP1S2	Adaptor-related protein complex 1, sigma 2 subunit	NM_003916.3	-11.5	-2.5	-2.1
CTNNAL1	Catenin (cadherin-associated protein), alpha-like 1	NM_003798.2	-13.9	-3.1	-2.1
DCBLD2	Discoidin, CUB and LCCL domain containing 2	NM_080927.3	-14.5	-3.1	-2.6
CAV1	Caveolin 1, caveolae protein, 22 kDa	NM_001753.3	-20.1	-3.3	-2.8

**Figure 4:**
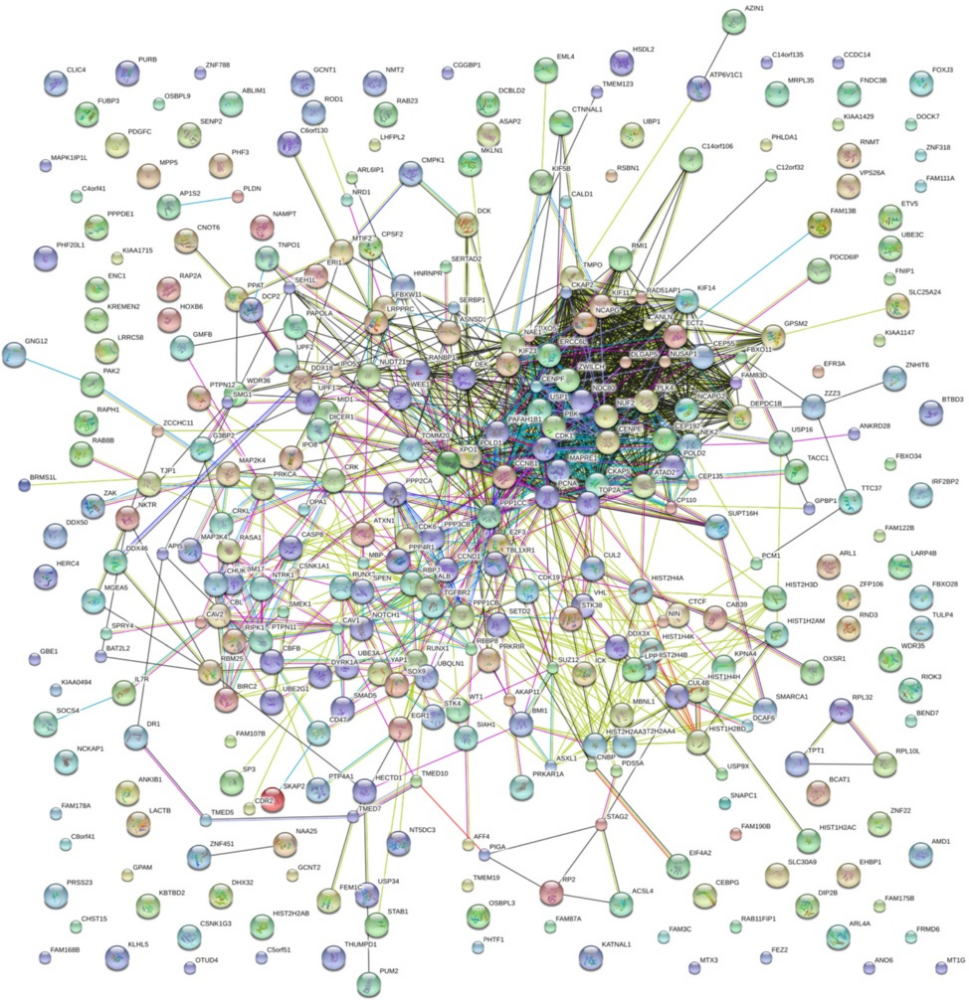
**Interaction network analysis of the differential response of the breast cancer cells to fever range hyperthermia reveals a strong node centered on mitotic cell cycle progression.** The list of 393 genes identified as differentially expressed in the breast cancer lines following fever range hyperthermia in the *H’* vs *H* comparison were queried using String 9.05. Lines illustrate known physical and functional associations derived from previously reported genomic context, high-throughput experiments, coexpression analysis, and Pubmed.

**Figure 5:**
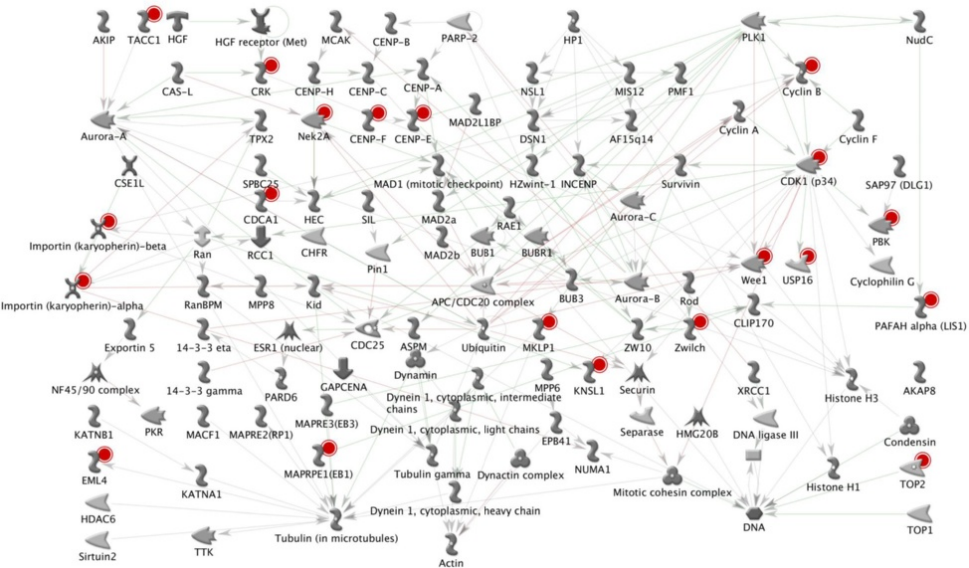
**Interrelationship between the mitotic regulators that differentiate the hyperthermic response of breast cancer cells from mammary epithelial cells.** Metacore analysis of the 393 genes that differentiate the hyperthermic response of breast cancer from mammary epithelial cells in the *H’* vs *H* comparison identified mitotic cell cycle progression (and 20 associated mitotic regulatory genes) as the primary differential gene networks. We used Metacore to identify the interrelationship of the known physical and functional associations between these 20 genes (*red markers*).

**Figure 6:**
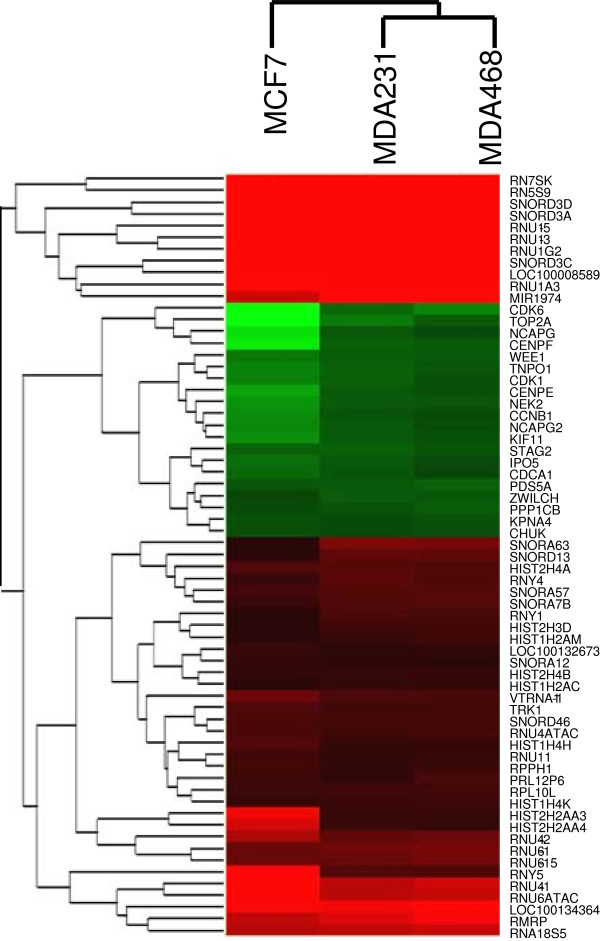
**Gene expression changes in histones, non-protein coding RNAs, and mitotic regulators differentiate the hyperthermic response of breast cancer cells from mammary epithelial cells.** Heatmap depicting the two-fold or greater changes in RNA expression levels (p < 0.05) for histone, non-protein coding RNA, and mitotic regulatory genes in the *H’* vs *H* comparison (*red?=?overexpressed, green?=?underexpressed*).

We performed computational analysis on the 393 genes using String software to identify interaction networks that might help reveal functional nodes indicative of the biological response of these cells to fever range hyperthermia. Our analysis uncovered a remarkably dense interaction node centered on genes involved in mitotic progression (Figure 4). We performed Metacore analysis on the list of 393 genes, confirming that mitotic cell cycle regulatory networks exclusively dominated the top statistically significant pathway maps (Table 4 lists the top 20 identified networks). Figure 5 illustrates Metacore’s analysis of the interrelationships of the identified mitotic regulatory genes including *STAG2, NEK2, KPNA4, IPO5, TNPO1, CCNB1, CDK1, CDK6, NCAPG, NCAPG2, TOP2A, NUF2, CENPE, CENPF, ZWILCH, PDS5A, WEE1, KIF11, CHUK,* and *PPP1CB*. Of the 393 genes that were differentially expressed between the breast cancer and mammary epithelial cells following *H’* to *H* analysis, approximately 80% of the top 60 most upregulated genes were histone clusters and non-protein coding RNAs such as small nucleolar-, ribosomal-, and micro-RNAs. These data cumulatively suggest that the selective disadvantage that breast cancer lines experience following mild hyperthermic shock may be due to an inability to correctly regulate their core biological processes and mitotic cell cycle machinery. The differential expression of genes involved in these processes for the *H’* vs *H* analysis is shown in Figure 6.

**Table 4:** **Top 20 significantly significant GeneGo pathway maps that are differentially expressed amongst all three breast cancer cell lines relative to the mammary epithelial line in the *H’ *** **vs *H *** **comparison**

**GeneGo Pathway**	**p-Value**
Mitosis	8.15e^-44^
Cell division	3.02e^-35^
Cell cycle	5.37e^-23^
Mitotic sister chromatid segregation	1.31e^-22^
Mitotic spindle organization	4.87e^-20^
Protein localization to kinetochore	1.54e^-19^
Chromosome segregation	4.85e^-16^
Establishment of mitotic spindle orientation	7.53e^-16^
Mitotic cell cycle	9.66e^-16^
Homologous chromosome segregation	3.30e^-13^
Mitotic cell cycle checkpoint	5.99e^-12^
Anaphase promoting complex-dependent degradation	2.27e^-10^
Mitotic cell cycle spindle assembly checkpoint	2.03e^-09^
Spindle assembly involved in mitosis	4.44e^-09^
Mitotic anaphase	6.97e^-09^
Microtubule-based movement	3.57e^-08^
Spindle organization	4.83e^-08^
Mitotic centrosome separation	7.75e^-08^
Spindle assembly	1.92e^-07^

### Altered expression of mitotic arrest genes differentiates the hyperthermic response of breast cancer cells from that of mammary epithelial cells

**Figure 7:**
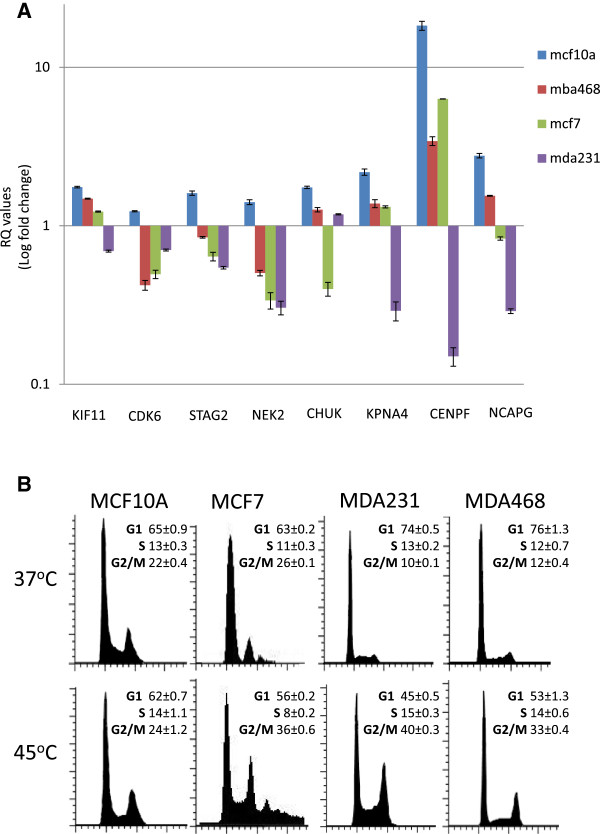
**Biological confirmation of gene expression analysis. (A)** qPCR analysis measuring the fold change of mitotic regulators following hyperthermia treatment. RQ values are represented as the hyperthermia-induced change in gene expression for each gene relative to the expression of the same gene in the normal temperature condition (RQ?=?1). The data shown are the median of at least 3 replicates, plus or minus the standard deviation, and presented in log scale. **(B)** The panel of mammary epithelial and breast cancer cells were grown under standard growth conditions or treated with 30 minutes fever range hyperthermic shock. Cells were harvested after 24 hours and cell cycle analysis was performed using flow cytometric detection of propidium iodide intensity.

Our microarray analysis strongly suggests that the inability of breast cancer cells to regulate their mitotic cell cycle machinery may be a major contributing factor to their selective disadvantage following hyperthermia. Therefore we independently tested the expression levels of a panel of mitotic regulators that were identified as differentially expressed in the *H’* vs *H* analysis. Quantitative real time PCR analysis of cDNA collected from the *H’* vs *H* treatments for the steady state mRNA levels of several genes with core processes related to mitosis including *KIF11, CDK6, STAG2, NEK2, CHUK, KPNA4, CENPF,* and *NCAPG* correlated well with our microarray data, revealing differential expression of these genes for each cell line in the hyperthermia treatment relative to the normal temperature (Figure 7A). A comparison of the qPCR and microarray data for each of these selected genes for the H’ to H comparisons is depicted in Table 5. To confirm the hyperthermia-induced mitotic defect in the breast cancer lines, we subjected all four cell lines to 30 minutes of fever range hyperthermia (*H* and *H’*) or normal control temperature (*C* and *C’*) and collected the cells after 24 hours for cell cycle analysis using flow cytometry. The cells were collected 24 hrs after treatment as this is sufficient time to see the phenotypic effects on the cell cycle that would be induced by altered RNA expression. Propidium iodide staining of cells from each condition clearly revealed that a G2/M phase accumulation as a common event across all three breast cancer lines following hyperthermia even after 24 hours following the treatment, but did not occur in the mammary epithelial lines (Figure 7B). Collectively, these data provide evidence to suggest that the selective disadvantage of breast cancer cells in response to hyperthermia could be due, in part, to altered regulation of mitotic machinery following heat shock.

**Table 5:** qPCR and microarray expression data for selected cell cycle genes in the H’ vs H comparison

**Gene Symbol**	**MCF7**		**MBA231**		**MBA468**	
	**qPCR**	**Array**	**qPCR**	**Array**	**qPCR**	**Array**
*KIF11*	-1.5	-5.2	-2.6	-3.3	-1.2	-3.1
*CDK6*	*-*2.4	-9.4	-1.7	-3.1	-3.0	-4.1
*STAG2*	-2.7	-3.0	-3.2	-2.9	-2.0	-2.5
*NEK2*	-4.7	-4.6	-1.7	-2.7	-2.8	-2.6
*CHUK*	-4.3	-2.3	-1.4	-2.2	-1.3	-2.3
*KPNA4*	-1.7	-2.4	-7.3	-2.2	-1.6	-2.4
*CENPF*	-2.9	-7.4	-183	-2.9	-5.4	-2.7
*NCAPG*	-3.5	-4.2	-9.3	-2.7	-1.9	-2.2

p?≤?0.05 for all values via Student’s t-test.

## Discussion

While hyperthermic treatment of tumors has been utilized since the time of the ancient Greeks and modern medicine has implemented hyperthermia as an adjuvant treatment in various settings, use of this technique has been marred with limitations including the inability to target heat to the tumor without collateral damage to the neighboring cells, homogenous heat dispersion throughout the entire tumor, and intrinsic problems with targeting undetectable micrometastases. In recent years, advances in nanoparticle-enabled thermal therapy hold the promise to overcome many of these issues, thus a strong interest in treatment of tumors with hyperthermia has been renewed. While it has been established for decades that normal tissues exhibit enhanced thermotolerance relative to cancer cells [1,2], the mechanisms controlling this are largely unknown. Studies on the heat shock response of cancer cells have revealed changes in apoptosis, cell cycle regulation, and cell structure/maintenance [3], yet very little has been reported critically comparing the heat shock responses of cancer cells to their non-diseased cellular counterparts. Thus it is currently unknown at the molecular level how thermotolerance is maintained in normal cells, but lost or deregulated in cancer cells. To address this, we utilized a genomics approach to address two areas: 1) identify the global transcriptional response to hyperthermia of a panel of breast cancer and mammary epithelial cells using a *C* vs *H* and *C’* vs *H’* analysis and 2) compare the hyperthermia-induced changes in global gene expression patterns of the breast cancer cell lines to the mammary epithelial cells using a *H’* vs *H* analysis. As a result of these studies, we identified several gene networks that reflect the hyperthermic response of breast cancer and mammary epithelial cells (including cell cycle, heat shock, survival/apoptosis, DNA damage and Rab/Ran regulation) and that clearly differentiate the response of breast cancer cells from that of mammary epithelial cells (including mitotic regulation and expression of histone and non-protein coding RNAs).

### Evaluation of the hyperthermic response of breast cancer and mammary epithelial cells

*C* vs *H* and *C’* vs *H’* comparative analysis of the gene expression profiles of each cell line revealed that the mammary epithelial cells responded to increased temperature distinctly from the breast cancer lines, with altered regulation of gene networks controlling DNA damage response, cell cycle progression, apoptosis, and heat shock characterizing the mammary epithelial cell response. In contrast, the three breast cancer lines commonly altered gene networks encoding Rab and Ran G-protein regulators in response to hyperthermia.

Arguably the most studied response of cells to hyperthermia is that of heat shock protein activation and expression and one might guess that heatshock-protein mediated responses are likely responsible for the selective disadvantage of solid tumors to fever range hyperthermia. Numerous cell stresses have been shown to induce heat shock proteins, which act as molecular chaperones inside cells to modulate thermotolerance and protect cells from stress-induced death [27-29]. MCF10A cells exhibited significantly increased expression of HSP90AA1, CARHSP1, HSPA12A and decreased expression of HSPB1, HSPBL2, HSPA6, and HSPA7 (*C* vs *H*), while the three breast cancer lines showed no significant 2-fold or greater alterations in the expression of these genes (*C’* vs *H’*). Despite this finding, we provide evidence that suggests the ability of mammary epithelial cells to properly modulate their heat shock response does not contribute to the selective disadvantage of breast cancer cells to hyperthermia. For instance, comparison of heat shock protein expression in the *H’* vs *H* analysis revealed no significant difference in the relative abundance of these heat shock protein genes regardless of the cell type. As elevated expression of heat shock proteins has been observed in various types of cancers [30-32], hyperthermic shock may simply bring the heat shock protein expression in MCF10A cells to the baseline levels in the breast cancer lines.

Hyperthermia has long been known as an effective radio- and chemo-sensitizing agent and it would be an attractive hypothesis that hyperthermia may impart a selective disadvantage to breast cancer cells via upregulation of DNA damage or reduction in its repair. Indeed, hyperthermia has been shown to induce chromosomal damage during S-phase [33] and inhibit homologous recombination repair via a heat shock protein/Brca1/2 pathway [34-36]. Furthermore, hyperthermia induces signaling pathways that overlap with those activated by ionizing radiation-induced DNA damage including histone H2Ax phosphorylation and enhanced ataxia-telangiectasia mutated protein (ATM) activity [37]. Analysis of *C* vs *H* and *C’* vs *H’* revealed a number of genes involved in DNA damage response whose expression was altered in the MCF10A cells, and similar changes were not observed in the three breast cancer lines. Despite this, no statistically significant changes in gene expression for these genes were observed in our *H’* vs *H* comparison, suggesting that (similar to the heat shock proteins) this pathway may not clearly distinguish the selective disadvantage of breast cancer cells to hyperthermia.

### Gene networks that distinguish the hyperthermic response of breast cancer cells from mammary epithelial cells

As our initial analysis compared only the heat shock response of each individual cell line relative to its transcriptional expression at normal growth temperature, we extended our analysis by directly comparing the transcriptional response of the *H’* vs *H* treatments to identify the unique gene networks that clearly differentiate the gene expression changes unique to the breast cancer cells following heat treatment. This comparative analysis identified cell cycle networks preferentially involved in mitotic progression as well as large scale changes in the expression of histones and non-protein coding RNAs as the major distinctions between the hyperthermic responses between the breast cancer lines and the MCF10A cells. 80% of the top 60 genes commonly expressed at higher levels in the three breast cancer lines following heat shock relative to the mammary epithelial line following heat shock were histones and non-coding RNA. This effect was due primarily to decreased expression of these genes in the MCF10A cells with no change or only a small upregulation in expression in the breast cancer lines, suggesting that mammary epithelial cells are repressing many of their core processes (chromatin condensation, transcription, translation, etc.) following hyperthermic shock, while the breast cancer cells may continue performing these processes as normal. Similar findings have been reported following other cellular stresses whereby oxidative damage significantly decreases the expression of histones and ribosomal proteins [38]. In addition to histone gene expression, heat shock induces an array of chromatin post-translational modifications. For instance, HSP70 has been shown to enhance the phosphorylation of histone H3 following heat shock [39], and histone variant H3.3 has been shown to stimulate heat shock induced HSP70 transcription [40], suggesting that heat shock response and histone activity are tightly regulated.

Our data revealed that a number of small nucleolar RNAs, which play key roles in ribosomal biosynthesis, were differentially regulated between the mammary epithelial and breast cancer cells following hyperthermia. Several small nucleolar RNAs are reportedly critical mediators of oxidative stress and their overexpression has been associated with reduced resistance to oxidative stress [41,42]. Small nucleolar RNAs have been shown to bind to the mature RNA of heat shock cognate protein (HSC70) [43] and inhibition of Hsp90 prevents the accumulation of U3 and U4 small nuclear ribonucleoproteins via a process that involves Pih1/Nop17 and R2Tp complexes [44-46].

Changes in cell cycle progression (particularly mitotic catastrophe) have been repeatedly shown to characterize the hyperthermic response of numerous cell types [47-49], though it is largely unstudied as to how cell cycle changes differ between normal and tumor cell lines following this treatment. Major distinctions in cell cycle networks involved in mitotic progression clearly distinguished the *H’* vs *H* analysis of our data. The genes that were differentially expressed at statistically significant levels included those involved in spindle assembly and chromosome separation, chromosome condensation in prometaphase, metaphase checkpoint, sister chromatid cohesion, and initiation of mitosis. Our confirmatory experiments using flow cytometry further revealed that hyperthermia treated breast cancer cells stalled in the G2/M phase of the cell cycle within 24 hours post-treatment, while the cell cycle profiles of heat-shocked mammary epithelial cells were similar to those grown at normal temperatures. Interestingly, it has been shown that cells vary in their susceptibility to heat in accordance to their phase in the cell cycle, with the highest heat sensitivity observed during mitosis due to damage to the mitotic apparatus, leading to inefficient mitosis and polyploidy. M- and S-phase arrested cells show increased susceptibility to heat-induced damage, while G1-phase cells are relatively heat resistance [50-53].

This study has been the first to shed light on the comparisons of transcriptome-level fever range hyperthermic responses of mammary epithelial cells to breast cancer cells. While this data points to a number of areas that potentially contribute to the selective advantage of normal breast epithelium over its malignant counterparts following hyperthermia, our studies were simplistic in that they utilized a cell culture monolayer system solely consisting of cells derived from normal or tumor breast tissue. We have gained solid insight into the responses of these particular cell types to fever range hyperthermia, however a tumor is a very complex entity. For instance, solid tumors are not only composed of the tumor cells, but also consist of endothelial, fibroblast, and immune cells which will each respond to hyperthermia in their own fashion and potentially affect the response of the tumor as a whole. Moreover, heterogenous heat distribution and dissipation due to a faulty tumor vascular system may induce uneven heating in the tumor itself, thus affecting some areas distinctly and differentially altering the tumor’s response to hyperthermia. Future studies should be undertaken to address these issues.

## Conclusion

Collectively, our data suggest that fever range hyperthermia affects breast cancer cells distinctly from mammary epithelial cells. These differences are largely attributed to alterations in the expression of genes involved in mitotic cell cycle progression, histones, and non-coding RNAs. Considering the hyperthermia induced G2/M cell cycle defects observed in the breast cancer cells but not the mammary epithelial cells, these data pose the question as to whether hyperthermia may function in a synergistic manner when combined with drugs that specifically target mitosis such as taxols and vinca alkaloid derivatives.

## Abbreviations

C: 37°C treatment of mammary epithelial cells; C’: 37°C of breast cancer cells; H: 45°C of mammary epithelial cells; H’: 45°C of breast cancer cells; RNA: Ribonucleic acid; DNA: Deoxyribonucleic acid; aRNA: Amplified RNA; cDNA: Complementary DNA; mRNA: Messenger RNA; G1: Gap 1; S: Synthesis; G2: Gap 2; M: Mitosis; RT PCR: Real time polymerase chain reaction.

## Competing interests

The authors declare that they have no competing interests.

## Authors’ contributions

CA contributed to experimental concept and design, cultured and treated cells, purified RNA, performed analysis and interpretation of microarray data, carried out network analysis, drafted and revised manuscript, gave final approval for version to be published. VK contributed to interpretation of network analysis, assisted with drafting and revising manuscript, gave final approval for version to be published. JS cultured and treated cells, purified RNA, drafting and revising manuscript, gave final approval for version to be published. AN performed flow cytometric cell cycle analysis and interpretation of data, gave final approval for version to be published. AA cultured cells, gave final approval for version to be published. RL contributed to experimental concept and design, drafted and revised manuscript, gave final approval for version to be published. CB contributed to concept and design, drafted and revised manuscript, gave final approval for version to be published. DM performed microarray experiments, performed bioinformatics analysis and interpretation of data, drafted and revised manuscript, gave final approval for version to be published. BB contributed to concept and design, acquisition of data, analysis and interpretation of data, drafted and revised manuscript, gave final approval for version to be published.

## Pre-publication history

The pre-publication history for this paper can be accessed here:

http://www.biomedcentral.com/1471-2407/14/81/prepub
